# Multi-cohort analysis of colorectal cancer metagenome identified altered bacteria across populations and universal bacterial markers

**DOI:** 10.1186/s40168-018-0451-2

**Published:** 2018-04-11

**Authors:** Zhenwei Dai, Olabisi Oluwabukola Coker, Geicho Nakatsu, William K. K. Wu, Liuyang Zhao, Zigui Chen, Francis K. L. Chan, Karsten Kristiansen, Joseph J. Y. Sung, Sunny Hei Wong, Jun Yu

**Affiliations:** 1 Institute of Digestive Disease and Department of Medicine and Therapeutics, State Key Laboratory of Digestive Disease, Li Ka Shing Institute of Health Sciences, Shatin, NT Hong Kong; 2 CUHK Shenzhen Research Institute, The Chinese University of Hong Kong, Pok Fu Lam, Hong Kong; 3 Department of Microbiology, Faculty of Medicine, The Chinese University of Hong Kong, Pok Fu Lam, Hong Kong; 4 Laboratory of Genomics and Molecular Biomedicine, Department of Biology, University of Copenhagen, Copenhagen, Denmark; 5 Institute of Metagenomics BGI-Shenzhen, Shenzhen, China

**Keywords:** Colorectal cancer, Microbiota, Diagnostic marker, Ecology

## Abstract

**Background:**

Alterations of gut microbiota are associated with colorectal cancer (CRC) in different populations and several bacterial species were found to contribute to the tumorigenesis. The potential use of gut microbes as markers for early diagnosis has also been reported. However, cohort specific noises may distort the structure of microbial dysbiosis in CRC and lead to inconsistent results among studies. In this regard, our study targeted at exploring changes in gut microbiota that are universal across populations at species level.

**Results:**

Based on the combined analysis of 526 metagenomic samples from Chinese, Austrian, American, and German and French cohorts, seven CRC-enriched bacteria (*Bacteroides fragilis*, *Fusobacterium nucleatum*, *Porphyromonas asaccharolytica*, *Parvimonas micra*, *Prevotella intermedia*, *Alistipes finegoldii*, and *Thermanaerovibrio acidaminovorans*) have been identified across populations. The seven enriched bacterial markers classified cases from controls with an area under the receiver-operating characteristics curve (AUC) of 0.80 across the different populations. Abundance correlation analysis demonstrated that CRC-enriched and CRC-depleted bacteria respectively formed their own mutualistic networks, in which the latter was disjointed in CRC. The CRC-enriched bacteria have been found to be correlated with lipopolysaccharide and energy biosynthetic pathways.

**Conclusions:**

Our study identified potential diagnostic bacterial markers that are robust across populations, indicating their potential universal use for non-invasive CRC diagnosis. We also elucidated the ecological networks and functional capacities of CRC-associated microbiota.

**Electronic supplementary material:**

The online version of this article (10.1186/s40168-018-0451-2) contains supplementary material, which is available to authorized users.

## Background

Colorectal cancer (CRC) is one of the most common cancers in the world with over 1 million cases diagnosed every year [1]. Many risk factors, including genetic, dietary, and other environmental factors contribute to CRC. The association of CRC with an altered gut microbiota has been studied in different populations, identifying bacteria such as *Fusobacterium nucleatum* and *Bacteroides fragilis* that are associated with tumorigenesis [2]. In this regard, *F. nucleatum* was found to modify the tumor immune microenvironment [3], while *B. fragilis* could produce DNA-damaging genotoxins in host cells [4]. *Prevotella* has been reported to be enriched in proximal colon cancer [5] and associated with interleukin (IL)-17-producing cells [6]. *Porphyromonas* has also been identified to be associated with CRC in different populations [7–9]. The potential use of these microbes as non-invasive biomarkers for the detection of CRC has been explored [10, 11]. However, studies from different populations may produce cohort-specific results. Furthermore, the gut microbiome is highly dynamic and influenced by dietary, xenobiotic, physiological, host genetics, and other factors [12], implying that results from metagenomic studies may not be applicable across different populations. Technically, metagenomic studies are also influenced by sample qualities, sequencing platforms, and the bioinformatic pipelines used for analysis [13]. These factors may result in heterogeneity and inconsistency among studies. Meta-analysis has an advantage of increasing statistical power over individual studies [12]. With a larger sample size, meta-analysis can pinpoint differences that are too small to be detected by single cohort studies while simultaneously considering population-specific characteristics [12]. By combining 16S rRNA gene sequence data sets from nine studies, Shah and colleagues recently identified a general composite microbial marker for CRC [14]. Nevertheless, interpretation of results generated by 16S rRNA sequencing may be limited by its low taxonomical and functional resolution. The use of shotgun metagenomics sequencing allows the identification of bacterial taxa to species level [14] and is useful for analyzing gut microbiota functions without reliance on prediction [15]. We have previously reported on metagenomic features shared by CRC patients of different ethnicities [16]. We extended this work by performing a comprehensive meta-analysis of shotgun metagenomic data acquired from CRC patients and control subjects of American, Austrian, Chinese, and German and French cohorts to achieve greater statistical power to investigate the association of gut microbiota with CRC.

## Results

### Microbiota composition across cohorts

**Fig. 1:**
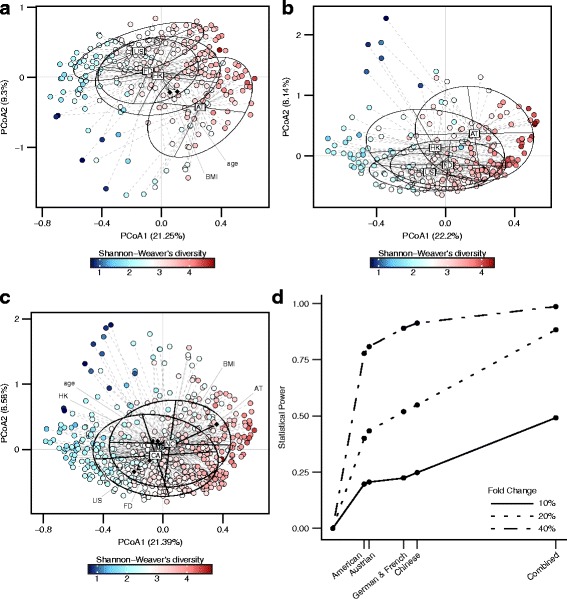
Microbial composition and statistical power difference across cohorts. **a–c** Principal coordinate analysis for control samples, CRC samples, and all samples, respectively. (CA, CRC; NC, negative control) The correlations between phenotypes and PCoAs are labeled with their corresponding coordinates. **d** Statistical power to detect differentially abundant bacteria of various fold change (fold change = 10, 20, and 40%) versus cohort sample size (number of control samples × number of case samples)

We accumulated shotgun metagenomic sequencing sequences from four cohorts (USA (USA), Austria (AT), China (HK), and Germany and France (FD)), including 271 controls and 255 CRC cases (demographic, clinical, and technical details are shown in Table 1). The sequences were curated, and we used Kraken v_0.10.5-beta for sequence classification and alignment. The Shannon diversity indexes were not significantly different between CRC cases and controls in USA, AT, and FD cohorts. Nevertheless, the diversity index decreased significantly in cases compared to controls in HK cohort (*p* = 0.045, Additional file 1: Figure S1A). Principal coordinate analysis based on Bray-Curtis dissimilarity index identified significant bacteria compositional difference among cohorts (PERMANOVA, in control samples, *p* < 1 × 10^−4^, Fig. 1a; in CRC samples, *p* < 1 × 10^−4^, Fig. 1b). We also found significant differences in overall bacterial composition between cases and controls (PERMANOVA, *p* < 1 × 10^−4^, Fig. 1c).

**Table 1:** Fecal samples’ demographic, clinical, and technical details

Cohort	Factor	Control	CRC	*P* value	Sample collection	Sequencing platform
Cohort C1 (American, 2016)	Sample size	52	48	NA	Prior to surgery and treatment	Sequencing Platform: Illumina Hiseq 2000/2500; Sequencing Target Depth: 5GB; read length: 100 bp
	Age	61.23(11.03)	60.96(13.56)	0.913		
	Gender	Male:37; Female:15	Male:35; Female:13	1		
	BMI	25.35(4.27)	24.90(4.29)	0.601		
Cohort C2 (Austrian, 2014)	Sample size	63	46	NA	Not available	
	Age	67.1(6.37)	67.1(10.91)	0.999		
	Gender	Male:37; Female:26	Male:28; Female:18	0.978		
	BMI	27.57(3.78)	26.50(3.53)	0.132		
Cohort C3 (Chinese, 2015)	Sample size	92	73	NA	No antibiotics and no invasive medical intervention for 3 months; no vegetarian diet; no history of cancer or inflammatory disease of intestine	
	Age	58.51(7.55)	65.90(10.61)	< 0.0001		
	Gender	Male:51; Female:41	Male:47; Female:26	0.316		
	BMI	23.87(3.31)	24.07(3.18)	0.697		
Cohort C4 (German and French, 2014)	Sample size	64	88	NA	No previous colon or rectal surgery, colorectal cancer, inflammatory, or infectious injuries of the intestine; no need for need for emergency colonoscopy	
	Age	58.75(12.96)	68.44(12.22)	0.007		
	Gender	Male:32; Female:32	Male:53; Female:35	0.276		
	BMI	24.72(3.19)	25.89(4.29)	0.056

### Bacteria differing in abundance between CRC cases and controls across cohorts

We performed simulation analysis to compare the statistical powers between the meta-analysis and single cohort studies. Our power simulation analysis showed an increased statistical power with the rank sum meta-analysis approach. Simulation analysis showed an estimated power of 0.88 with a bacterial abundance change of 20%. This shows an advantage over single-cohort studies with an approximate power of 0.5 at abundance fold change of 20% (Fig. 1d).

**Fig. 2:**
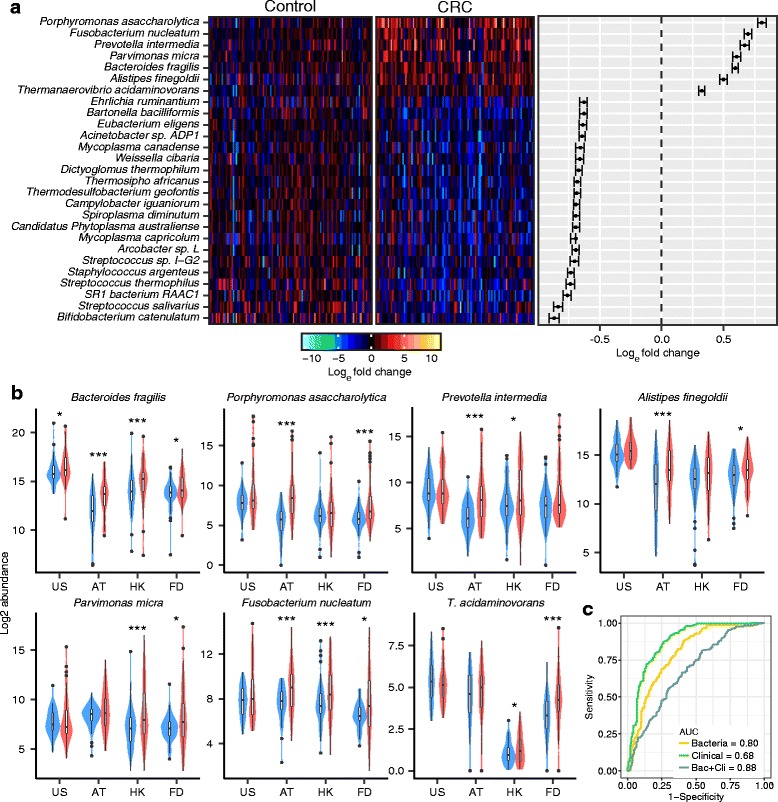
Differentially abundant bacteria in CRC across cohorts. **a** Left panel; abundance of 7 CRC-enriched species and 20 CRC-depleted species with largest fold change. The bacteria abundance was normalized to natural log fold change relative to abundance median of control samples. Right panel; confidence interval for individual pair fold change. The confidence intervals were calculated based on Wilcoxon signed-rank test. **b** Violin graph for the abundance of the 7 CRC-enriched bacteria in different cohorts. Abundance change significance within individual cohort is labeled with * (*P* < 0.05, *P < 0.01, **P < 0.001, ***). **c** Prediction power of 7 CRC-enriched bacteria with SVM model

We applied this meta-analysis approach to identify bacterial species that exhibited differential abundance in CRC compared to controls across all the four cohorts. After excluding bacteria showing ‘divergent directional changes,’ 994 species were obtained for further analysis (Additional file 2: Text). Using the rank sum method, we identified 7 enriched species and 62 depleted species in CRC cases compared to controls (Fig. 2a). We demonstrated that the significance levels (pfp) of the 69 different abundant species were not affected by our filtering pipeline (Additional file 3: Table S14). These results were validated with another independent pipeline, MetaPhlan [17] (Additional file 4: Table S4). The seven CRC-enriched species included *Bacteroides fragilis*, *Fusobacterium nucleatum*, *Porphyromonas asaccharolytica*, *Parvimonas micra*, *Prevotella intermedia*, *Alistipes finegoldii*, and *Thermanaerovibrio acidaminovorans.*

Among the seven CRC-enriched species, *Bacteroides fragilis* was observed to be consistently enriched across all four cohorts (Fig. 2b), whereas five bacteria showed significant changes in abundance in two of the four cohorts (*P. asaccharolytica*, *P. micra*, *P. intermedia*, *A. finegoldii*, and *T. acidaminovorans*). Five bacterial species among the 62 CRC depleted bacteria have been reported to confer health benefits, including *Clostridium butyricum* [18, 19], *Streptococcus salivarius* [20, 21], *Streptococcus thermophilus* [22], *Carnobacterium maltaromaticum* [23], and *Lactobacillus gallinarum* [24]*.* To identify whether the 69 CRC-associated bacteria correlated with CRC progression, we investigated their abundance difference between early- and late-stage CRC (Additional file 5: Table S1). Three species showed decreasing trends across control, early- and late-stage CRC (*Streptococcus sp. I-G2*, *Shewanella woodyi*, *Mycoplasma penetrans*, Additional file 6: Figure S5).

### Classification of CRC cases and controls with bacterial markers

To classify CRC cases from controls based on bacterial composition, the seven CRC-enriched bacterial species were fitted into a support vector machine (SVM) model with radial kernel [25]. We obtained areas under the receiver-operating curve (AUCs) of 0.83, 0.87, 0.84, and 0.82, respectively, for USA, AT, HK, and FD cohorts (Additional file 7: Figure S3B). As we used a SVM model with 10-fold cross validation, it achieved average AUC of 0.75 on the testing fold (Additional file 8: Figure S12A). The overall AUC was 0.80 for the combined population, and this performance was not significantly skewed by a single cohort (Additional file 7: Figure S3A). We further evaluated the classification power of bacteria markers by leaving-one-cohort-out approach. We leaved one cohort as the validation samples at each time and used the three cohorts left to select markers and train the SVM model. Our model achieved an average AUC at 0.73 on the validation samples (Additional file 8: Figure S12B). After the inclusion of clinical phenotype information, namely, age, gender, and body mass index (BMI), the overall AUC increased to 0.88 (Fig. 2c). Optimal F1 scores (harmonic mean of recall and precision) could reach 0.67 and 0.80, respectively using bacteria markers alone and combining clinical phenotype information (Additional file 9: Figure S4A, Additional file 10: Table S13 and Additional file 11: Table S15). Additionally, the potential of using the seven bacterial markers for diagnosis of early-stage CRC was evaluated. We calculated the significance of available bacterial abundance changes in AT, HK, and FD cohorts (cancer stage information is missing in USA). The results showed significant abundance changes for the seven CRC-enriched species in three cohorts, indicating that data from the three cohorts (besides cohort USA) were sufficiently informative for the stage-specific analysis (Additional file 12: Figure S6A). Using the SVM model, the seven CRC-enriched species classified early-stage CRC patients from controls with AUCs of 0.84, 0.82, 0.84 in AT, HK, and FD cohorts, respectively (Additional file 12: Figure S6B), suggesting an outstanding classification performance between early-stage CRC cases and controls.

### Correlations between CRC-related bacterial species

**Fig. 3:**
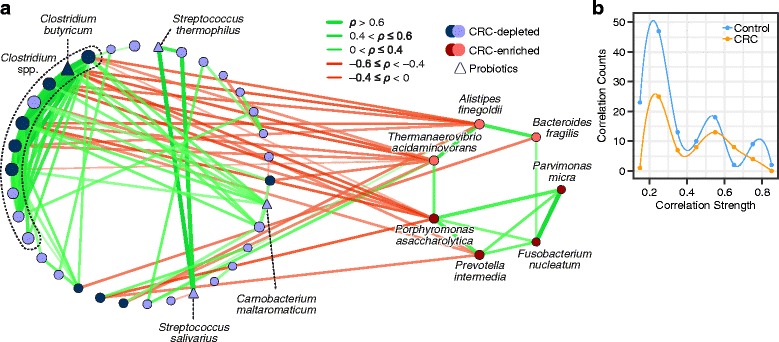
Meta-analysis of correlations among CRC-associated bacteria. **a** Correlation between the 69 CRC differentially abundant bacteria in CRC samples. Nodes having correlations between circles were labeled with dark blue, and the four CRC-enriched oral species were labeled with dark red. Five commensal bacterial species were denoted with triangle shape nodes. The size of the nodes is proportional to their corresponding centrality. Node attributes are included in Additional file 28: Table S3. **b** Comparison of the correlation network between CRC-depleted bacteria in control and CRC showing the mid-points of histogram bars. Cubic spline was used to connect the points

To gain insights into the bacteria-bacteria interactions from an ecological perspective, we further investigated the correlations between the CRC-enriched and CRC-depleted bacteria based on SparCC algorithm. The average correlation strength across the populations was also estimated (see “Methods”). We observed that the enriched and depleted bacteria, respectively, formed their own mutualistic networks that were negatively correlated with each other (Additional file 13: Figure S7). Interestingly, the number of significant correlation pairs and correlation strengths among CRC-depleted bacteria were higher in controls than in CRC cases (difference of significant correlation proportions: *p* = 6.4 × 10^−5^; difference of correlation strength: *p* = 2.6 × 10^−8^) (Fig. 3b). Most of the correlations between CRC-enriched and CRC-depleted bacteria were negative. The seven CRC-enriched bacteria were more closely correlated in early-stage than late-stage CRC, while the correlation networks between them was disrupted in late-stage CRC (Additional file 14: Figure S9C, correlation strength: *p* = 0.013). With weighted degree centrality, we found that *Clostridium* species (Additional file 15: Table S2) had the highest centralities in the network. These central species may play a pivotal role in the network, supported by analyses of the global efficiency and weighted network connectance (Additional file 16: Figure S8). Compared with the removal of random nodes, the network connectivity decreased very sharply when nodes of *Clostridium* species were removed.

### Functional gene families associated with CRC-enriched and CRC-depleted bacteria

**Fig. 4:**
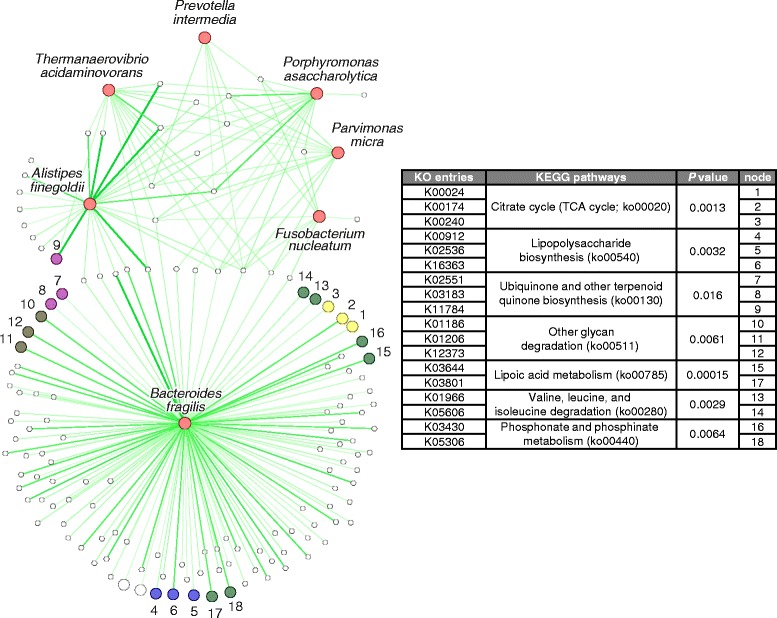
Correlation network between CRC-enriched bacteria and KO categories. Nodes with the same color share the same CRC-enriched pathway. Seven KEGG pathways that were enriched in CRC and involve CRC-enriched bacteria correlated KO categories are listed. The corresponding adjusted *P* values of the abundance change from control to CRC are also provided. Correlation details are attached in Additional file 29: Table S8 and Additional file 27: Table S9

The metagenome sequences were mapped to UniRef database and grouped into 10,675 gene ontology (GO) and 8695 KEGG ontology (KO) categories with HUMAnN2. The GO/KO reads were normalized to relative abundance (copy per million units) for comparison. A total of 311 GO categories and 217 KO categories were identified to be enriched in CRC (FDR < 0.05); whereas 31 GO categories and 74 KO categories were depleted in CRC (Additional file 17: Table S6 and Additional file 18: Table S7). We investigated the correlation between the seven CRC-enriched species and GO/KO categories with Spearman’s correlation, and identified 167 GO categories and 143 KO categories that have significant positive correlations (FDR < 0.05) with the CRC-enriched bacteria (Fig. 4, Additional file 16: Figure S8 and Additional file 14: Figure S9). We defined these GO/KO categories as CRC-enriched bacteria correlated GO/KO categories.

To investigate the functional pathways involved, we mapped the KO categories to the KEGG pathway. KO categories that were involved in the same pathway were treated as correlated KO categories. We observed 45 KEGG pathways involving at least two CRC enriched-bacteria correlated KO categories (Additional file 19: Table S10). To identify the pathways shared by multiple KO categories in association with cancer development, we found seven KEGG pathways whose overall abundance were enriched in CRC, namely, ko00020 (Citrate cycle (TCA cycle)), ko00540 (Lipopolysaccharide biosynthesis), ko00130 (ubiquinone and other terpenoid-quinone biosynthesis), ko00785 (lipoic acid metabolism), ko00280 (valine, leucine, and isoleucine degradation), ko00440 (phosphonate and phosphinatemetabolism), and ko00511 (other glycan degradation) (Fig. 4). Some commensal bacterial species were found to be correlated with CRC-depleted GO/KO categories; *Clostridium butyricum* and *Carnobacterium maltaromaticum* were strongly correlated with GO0051606 (stimulus detection; *ρ*=0.56 and 0.51, respectively) while *Streptococcus salivarius* and *S. thermophiles* were correlated with K07104 (catechol 2,3-dioxygenase; *ρ*=0.78 and 0.67, respectively) and K07570 (general stress protein 13; *ρ*=0.74 and 0.66, respectively) (Additional file 20: Figure S11).

## Discussion

In this study, we performed the first comprehensive meta-analysis of shotgun metagenomics on CRC. We assembled samples from three different continents and four different ethnic cohorts including a large number of CRC cases and control fecal samples. We identified species-level bacterial markers that were enriched and depleted in CRC across cohorts with a robust statistical method, rank sum, which was a model-free approach suitable for handling non-normal data. Further analysis provided inferences about the correlations between the bacterial markers and their possible functional roles. This study shows how meta-analysis of shotgun metagenomics data can provide useful biological information by identifying biomarkers with higher statistical power.

Heterogeneity of the microbiota was observed across different cohorts. From the principal component analysis results, we observed very significant *P* values for the difference in microbial composition among the populations. This observation is consistent with previous studies showing effects of ethnicity and technical differences on gut microbiota [26–28] and highlights the need for combined analysis.

The meta-analysis approach has been used to evaluate and combine results of comparable studies [29] with major advantages of increasing statistical power and improving estimates of effect size in association studies [30]. Our meta-analysis, using the rank sum method, identified seven bacterial markers that were CRC-enriched across four cohorts. Our results are supported by the fact that six out of the seven identified CRC-enriched bacteria, namely, *P. asaccharolytica*, *F. nucleatum*, *B. fragilis*, *P. intermedia*, *P. micra*, and *A. finegoldii* have been reported previously to be associated with CRC [5] in some but not all populations. *P. micra* was found to be significantly enriched in CRC and strongly correlated with *F. nucleatum* in Chinese cohort [16]. Our results suggest that *P. micra* can be universally associated with CRC across the cohorts. The *Alistipes* genus was found to be associated with CRC in a previous study [31], while *A. finegoldii* was isolated from the blood culture of CRC patient [32]. These observations support their roles in colorectal carcinogenesis. *Prevotella* was found to be enriched in proximal colon cancer [5], despite the controversial results reported in another study [33]. From our analysis, *P. intermedia* was clearly an enriched species in CRC, after combining the information from four cohorts. Interestingly, among the seven CRC-enriched species, four are oral bacteria (*P. asaccharolytica*, *F. nucleatum*, *P. intermedia*, and *P. micra*). Though the correlation between oral bacteria and CRC has been reported [34], our results suggest such a relationship exists across populations.

Our results also show a good performance of the seven CRC-enriched bacteria in classifying CRC from controls across cohorts. Population-specific variations may limit the classification performance of individual bacterial markers. For example, *F. nucleatum* has been used for CRC diagnosis in our previous study (AUC = 0.85) [10], but it is unknown whether it may be discriminative for other populations (Fig. 2b). However, through meta-analysis, we found a set of bacterial markers that was robust to population variations, which was exemplified by the performance of the seven bacterial markers achieving AUCs of at least 0.80 across all four populations. Our study also has the advantage of an overall balanced case-control sample size, which, under similar AUCs, usually provides a higher *F*_1_ score compared with a cohort with imbalanced case-control sample size due to accuracy paradox. As shown in our simulation analysis, when the sample size was imbalanced with a much smaller size in CRC cases than controls, the optimal *F*_1_ score was much smaller. Our result suggests that the prudent addition of markers may improve diagnostic performance and emphasize the advantage of meta-analysis in identifying markers applicable to multiple populations.

Besides the CRC-enriched bacteria, we identified five bacterial species previously reported to confer health benefits among the 62 CRC-depleted bacteria. The protective role of the bacteria during the colorectal carcinogenesis has not been thoroughly studied. *Clostridium butyricum*, identified in this study, was previously found to promote the apoptosis of CRC cells and inhibit intestinal tumorigenesis in mice [35]. This supports our finding of negative association between these bacteria and CRC (Fig. 3), and suggest that they may potentially act as probiotics to inhibit CRC progression.

In addition to bacterial abundance, changes in bacterial correlations could partially explain colorectal tumorigenesis. A closely correlated network between the CRC-depleted bacteria which may play a role in stabilizing the gut microbiota was observed. Its disruption may potentially contribute to colorectal carcinogenesis. On the other hand, the negative correlations between CRC-enriched and -depleted bacteria suggest the possibility of reciprocally antagonistic effects between them. The depletion of some commensal bacteria could reduce the suppressive effects on CRC-enriched bacteria, contributing to their enrichment. We also observed some interesting changes of the bacterial correlation pairs. Two oral bacteria, *P. micra* and *F. nucleatum*, were proposed to be strongly correlated in CRC in a previous study [16], which was also validated in our meta-analysis. This correlation was observed not to be significant in control samples, indicating that the CRC environment may be an important factor for the formation of this correlation and the two species may function cooperatively in CRC.

The metabolic functions of microbiota associated with CRC remain largely unknown. Our analysis revealed some functional shifts related to bacterial enrichments. Bacterial changes in CRC may result in the alteration of some functional gene-families and pathways to contribute to colorectal carcinogenesis. According to our results, over 50% of the CRC-enriched GO/KO categories significantly correlated with the CRC-enriched bacteria, suggesting their non-negligible contribution to the overall metabolic functionality in CRC. Interestingly, most of the CRC-enriched GO/KO categories that correlated with *B. fragilis* were discrete from other CRC-enriched bacteria, suggesting that *B. fragilis* may function independently in CRC (Fig. 4, Additional file 21: Figure S10). We also found some CRC-associated pathways identified by previous researchers. This included the pathway related to lipopolysaccharide (LPS), a gram-negative bacterial antigen that can induce toll-like receptor 4 signaling and promote cell survival and proliferation in CRC [36]. LPS has been shown to enhance cell migration in esophageal cancer cell line [37]. Our observation of the enrichment of this pathway in CRC and its correlation with CRC-enriched bacteria suggests its role in CRC and supports previous observations. Other bacterial functions involving biosynthetic pathways, metabolism of cofactors and vitamins and energy production pathways positively correlated with the CRC-enriched bacteria. These pathways may serve as alternative bioenergetic sources for metabolically stressed cancer cells [38].

Though we considered possible confounding factors, namely age, gender and BMI, other potential confounders such as tumor location, comorbidities, and cancer status were not included in this study. We used a filtering pipeline to remove bacteria showing divergent abundance changes (Additional file 22: Figure S13). While this may have led to the missing of some interesting species, this approach should have minimized the false positive discovery rate. It is likely that the seven identified species are genuinely related to CRC. Statistically, the sparsity of the bacterial abundance data makes it difficult to select features accurately. Additionally, the correlation and functional analysis were performed based on bacterial abundance and sequencing data. The difference between the mucosal and fecal microbiota may lead to the inconsistency of our findings and the real changes of microbiota in gut during the colorectal carcinogenesis. Despite these limitations, our study aggregated and uniformly analyzed deep-sequenced microbial fecal samples from diverse populations and found bacterial species that were consistently enriched in CRC across cohorts. The shotgun metagenome sequences used in this study equipped us with species-level identification and allowed analyses on bacterial correlations and metabolic functions. Importantly, our study provides directions for further research on the CRC microbiota.

## Conclusions

With the advancement in next generation sequencing technique, more metagenomic sequencing data sets are available, providing higher resolution of bacterial sequences. Though previous studies have widely reported the association between microbiota and CRC, it is essential to determine bacterial markers that are robust to population specific characteristics. Our study identified a group of bacteria that is consistently associated with CRC and shows potential in the diagnosis of CRC across multiple populations despite technical and biological variations. Universal ecological and functional shifts related to bacterial enrichment and depletion were also revealed, providing directions for further research on the potential functional involvements of the gut microbiota in contributing to colorectal tumorigenesis.

Future meta-analysis of gut microbiota dysbiosis in CRC should include more high quality metagenomic sequences especially from ethnic groups seldom covered by previous studies, such as African and south-east Asian, to provide higher statistical power and build up a more complete overview of CRC associated microbial dysbiosis. New methods for analyzing microbiome compositional data are also indispensable. Present analysis of the microbiome data treats every taxon independently, while the correlation structure between taxa has not been well incorporated, which may lead to high false discovery rate. With more available data sets and more powerful statistical analysis methods, better microbial CRC diagnostic tools and deeper understanding of microbial functions in colorectal carcinogenesis are obtainable.

## Methods

### Sequence curation and quality control

Raw sequences from CRC fecal metagenomics studies published from year 2014 to 2016 and with similar sequencing depths and target read lengths were retrieved from NCBI database [16, 31, 39, 40]. A total of 255 CRC patients and 271 controls from four cohorts (USA (US), Austria (AT), China (HK), and Germany & France (FD)) were included in this meta-analysis. Whole-genome shotgun sequencing of the samples from all cohorts was carried out on Illumina HiSeq 2000/2500 (Illumina, San Diego, USA) platform with similar sequencing depths (read length 100 bp, target sequencing depth 5 GB). Trimmomatic v_0.36 was used to remove low quality sequences. Human sequences were removed after alignment with reference genome (hg38 database [41]) using Bowtie2 v_2.2.9, with default settings. Kraken v_0.10.5-beta was then used to for taxonomic classification of unmapped microbial reads [42]. By the default setting of Kraken, only complete genome sequences were included as the reference genome database to reduce the errors from contaminants [42]. Since our project focused on bacteria community, we only included the sequences whose lowest common ancestor can be aligned to bacteria database. In HK and FD cohorts, we removed one sample respectively with low bacterial read count compared with other samples (removed samples’ bacteria read count = 57,547 and 165,287). Species level read counts were rarefied to the minimum read counts in selected samples of each cohort, namely; 2,419,973, 1,596,424, 1,222,507 and 856,204 per sample, respectively, in US, AT, HK and FD cohorts to reduce the effects of uneven sampling in each cohort. Because the real sequencing depth varied across the cohorts, though their target sequencing depths were the same, we did not rarefy all the samples to the same read counts to maintain this difference. This approach gave us larger read counts in US, AT and HK cohorts, which could reduce the variance of the estimated relative abundance (Var(*y*_*ij*_/*y*_*i*+_) decreases provided a larger *y*_*i*+_). Principal coordinates analysis was used with Bray-Curtis dissimilarity matrix to visualize microbiota composition.

### Meta-analysis of differentially abundant bacteria

We performed combined analyses on bacteria showing concordant changes in the individual study cohorts. We excluded a bacterial candidate from the analysis, if it exhibited significant contradictory abundance change directions among different cohorts. In our project, we gave a point estimation for the bacteria abundance change direction using the median. We removed species showing a balanced discrepancy in abundance changes in the four cohorts (positive abundance changes in two cohorts and negative abundance changes in two other cohorts), or species showing significantly discrepant change in one or more cohorts. Statistical significance was defined by *p* < 0.05 by the Mann-Whitney *U* test (see Additional file 2: Text for details). Considering the non-normality and over-dispersion of the microbial composition data, the non-parametric rank sum method was used for meta-analysis [43–45]. To control the confounding effects, any bacterial taxa whose abundance was significantly associated with the confounding variable was adjusted for the confounder using linear regression: log(*y*_*i*_) = *β*_0_ + *X*_*i*_ ∗ *β*_1_, *i* = 1, 2, …, *n*; where *y*_*i*_ is the abundance of sample *i* and *X*_*i*_ is the value of confounding variable of sample *i*. The adjusted bacteria abundance has the form *y*_*i*_^′^ = *y*_*i*_ ∗ exp(−*X*_*i*_ ∗ *β*_1_). With this approach, the effect of the confounder on the fold change was adjusted: $\frac{{y_i}^{\prime }}{{y_j}^{\prime }}=\frac{y_i}{y_j}\ast \exp \left(-\left({X}_i-{X}_j\right)\ast {\beta}_1\right)$. Species with estimated percentage of false-positives (pfp) smaller than 0.01 were considered significant. The significance of bacterial abundance change in the individual cohort was estimated using conditioned Mann-Whitney test with COIN package in R. Age was observed to be a confounding factor in HK and FD cohorts (Table 1). Since age is continuous, we divided the ages into 4 groups (Additional file 23: Figure S2) and treated the age groups as the conditioned variable for the test.

### Evaluation of statistical power

To evaluate the statistical power of rank sum meta-analysis method, we simulated microbial composition data following Dirichlet-multinomial distribution [46]. Most of the species were assumed to have similar relative abundance in CRC and control. Only a small proportion of the species (50 species) were simulated to be associated with CRC. We applied rank sum on the simulated microbial composition data and selected the top 50 species with the smallest rank sum statistics as bacteria exhibiting differential abundance. Among the 50 species, the proportion of real differentially abundant bacteria in the simulation data was used as an estimation of the statistical power. The statistical powers were compared under the fold change of 10%, 20%, and 40%.

### Correlation and network analyses

Correlations between species were calculated with the SparCC algorithm, a robust method for correlation inference of sparse compositional data [47]. We calculated the correlation separately in the four cohorts. The overall correlation across the cohorts was estimated using Hedges and colleagues’ method, where the correlations were first taken as Fisher’s *r*-to-*Z* transformation and then, calculated through weighted average of the transformed scores [48, 49]. Only significant correlations with *P* value < 0.05 were included in the downstream analysis. Correlation estimation was implemented with package ‘metacor’ in R, and the network was visualized with Cytoscape v_3.4.0. The centrality of nodes in the correlation network was evaluated by weighted degree centrality using correlation strength as the weight. Network connectivity was measured by global efficiency and weighted network connectance, where the global efficiency is defined as ${E}_{\mathrm{global}}(G)=\frac{E(G)}{E\left({G}^{\mathrm{ideal}}\right)}=\frac{2}{n\left(n-1\right)}\ast \sum \limits_{i\ne j\in \mathrm{G}}\frac{1}{d\left(i,j\right)}$ (*d*(*i*, *j*) denotes distance between vertex *i* and *j*; if vertex *i* and *j* are not connected, $\frac{1}{d\left(i,j\right)}$ is defined as 0; *n* is the number vertices in the network; in our case, *E*(*G*^ideal^)=1); $\mathrm{weigthed}\ \mathrm{network}\ \mathrm{connectance}=\frac{\sum \limits_{i\ne j}w\left(i,j\right)}{n^2}$ (*w*(*i*, *j*) denoted the weight of the edge between vertex *i* and *j*).

### Functional analysis

We processed HMP Unified Metabolic Analysis Network (HUMAnN2) to determine the abundance of gene families [50]. MetaPhlAn2 and ChocoPhlAn pangenome database were used for functional profiling. Gene families determined by UniRef were mapped to Kyoto Encyclopedia of Genes and Genomes (KEGG) Orthogroups (KO and Gene Ontology (GO) database and were grouped into functional categories. The conditioned Mann-Whitney test estimated the *P* values for the abundance change of the KO/GO categories from control to CRC in each cohort and the MaxP method was used to determine the overall f for abundance change [51]. Spearman correlation was applied to estimate association strengths in determining the relationship between bacteria and KO/GO categories.

## Additional files

Additional file 1: **Figure S1.** (A) Shannon diversities of control and CRC samples in each cohort. (B) Combining the normalized Shannon diversity of all the control and CRC samples. The Shannon diversity was normalized to mean = 0 and standard deviation = 1. (PDF 606 kb)

Additional file 2: Text Removing species with divergent abundance change directions. (DOCX 14 kb)

Additional file 3: **Table S14.** Significance of the abundance changes with rank sum test. (XLSX 12 kb)

Additional file 4: **Table S4.** Correlation between differentially abundant bacteria in early CRC. (XLSX 21 kb)

Additional file 5: **Table S1.** (DOCX 14 kb)

Additional file 6: **Figure S5.** Boxplot of the three species whose abundance significantly decreased between control, early-, and late-stage CRC (*P* < 0.05, *P < 0.01, **P < 0.001, ***). (PDF 342 kb)

Additional file 7: **Figure S3.** (A) Prediction performance of seven CRC-enriched bacteria using a single model. (B) Prediction performance of the optimized the model of the individual cohort. (PDF 524 kb)

Additional file 8: **Figure S12.** (A) Prediction performance of the SVM model on the testing folds using 10-fold cross validation. (B) Prediction performance of the SVM model on the testing cohort using ‘leave-one-cohort-out’ approach. (PDF 125 kb)

Additional file 9: **Figure S4.** (A) The precision and its corresponding recall index under different cut-off. The optimal F1 score was also provided. (B–C) With 271 controls and 30 cases, simulate probabilities with AUC≈0.80. Panel B shows a simulated precision and recall graph. Panel shows histogram of the optimal F1 scores with 500 simulations. (PDF 213 kb)

Additional file 10: **Table S13.** (XLSX 46 kb)

Additional file 11: **Table S15.** Markers selected by leave-one-cohort-out. (XLSX 9 kb)

Additional file 12: **Figure S6.** (A) The table includes pfp of the seven CRC-enriched bacteria using all the four cohorts and three cohorts besides USA cohort. (B) Prediction performance of using seven CRC-enriched bacteria to classify early-stage CRC from control. (PDF 900 kb)

Additional file 13: **Figure S7.** (A) Correlation between the 69 CRC differentially abundant bacteria on CRC samples. The left circle includes the CRC-depleted bacteria and the right includes the CRC-enriched bacteria. (B) The correlation between the 69 CRC differentially abundant bacteria in control. Node attributes are attached in Additional file 15: Table S2 and Additional file 28: Table S3. (PDF 334 kb)

Additional file 14: **Figure S9.** (A)–(B) Correlations between the 69 differentially abundant bacteria in early- and late-stage CRC. Node attributes are attached in Additional file 4: Table S4 and Additional file 24: Table S5. (C) Comparison between the correlation networks among CRC-enriched bacteria in early- and late-stage CRC. (PDF 927 kb)

Additional file 15: **Table S2.** Correlation between differentially abundant bacteria in Control. (XLSX 22 kb)

Additional file 16: **Figure S8.** Connectivity of the correlation network between the CRC-depleted bacteria in control samples. (PDF 367 kb)

Additional file 17: **Table S6.** Differentially Abundant GO categories. (XLSX 17 kb)

Additional file 18: **Table S7.** Differentially Abundant KO categories. (XLSX 15 kb)

Additional file 19: **Table S10.** Correlation between bacteria correlated-CRC-enriched KO categories. (XLSX 22 kb)

Additional file 20: **Figure S11.** Correlation network between CRC-depleted bacteria and CRC-depleted GO/KO categories (correlations with *ρ* > 0.5 were labeled in the figure). Node attributes are attached in Additional file 25: Table S11 and Additional file 26: Table S12. (PDF 3924 kb)

Additional file 21: **Figure S10.** Correlation network between CRC-enriched bacteria and CRC-enriched GO categories. Node attributes are attached in Additional file 27: Table S9. (PDF 376 kb)

Additional file 22: **Figure S13.** Null distribution of the number of species left by removing the low abundant species, species missing in any cohort, and species with ‘divergent abundance change directions’. (PDF 117 kb)

Additional file 23: **Figure S2.** Stratification of age in HK and FD cohorts. Conditioned Mann-Whitney *U* test was performed conditioned on the age strata. (PDF 214 kb)

Additional file 24: **Table S5.** Correlation between differentially abundant bacteria in late CRC. (XLSX 21 kb)

Additional file 25: **Table S9.** Node attributes of the correlation network between CRC-enriched bacteria and GO categories. (XLSX 171 kb)

Additional file 26: **Table S11.** Correlation network between the CRC-depleted bacteria and KO categories. (XLSX 81 kb)

Additional file 27: **Table S12.** Correlation network between the CRC depleted bacteria and GO categories. (XLSX 29 kb)

Additional file 28: **Table S3.** Correlation between differentially abundant bacteria in CRC. (XLSX 19 kb)

Additional file 29: **Table S8.** Significant correlations between CRC-enriched bacteria and KO categories. (XLSX 28 kb)

## Abbreviations

AUC: Area under the receiver-operating characteristics curve
BMI: Body mass index
CRC: Colorectal cancer
FDR: False discovery rate
GO: Gene Ontology
HUMAnN2: HMP Unified Metabolic Analysis Network
KO: Kyoto Encyclopedia of Genes and Genomes (KEGG) Orthogroups
PC: Principal component
PCoA: Principal coordinates analysis
SVM: Support vector machine
