# Supplementary figures and images for: Multi-cohort analysis of colorectal cancer metagenome identified altered bacteria across populations and universal bacterial markers
Source: Microbiome. 2018 Apr 11;6:70. doi: 10.1186/s40168-018-0451-2 (PMC5896039; doi:10.1186/s40168-018-0451-2)

**A**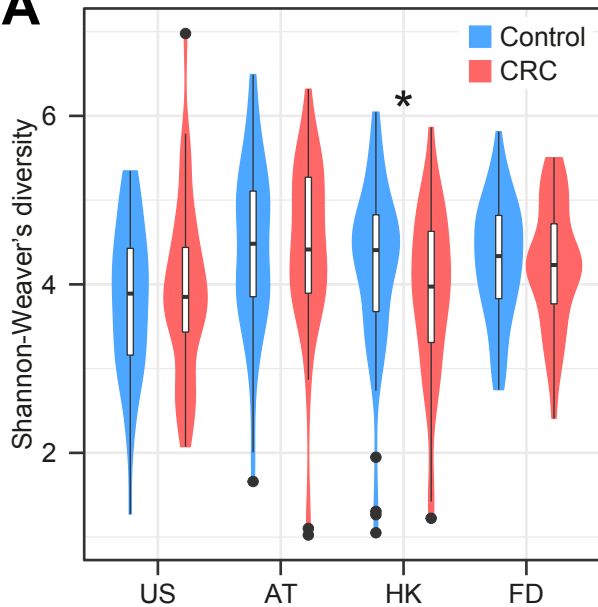**B**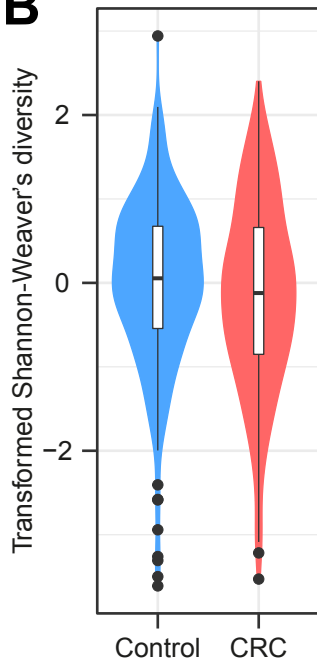

Supplement: Supplementary file 1 — Figure S1. (A) Shannon diversities of control and CRC samples in each cohort. (B) Combining the normalized Shannon diversity of all the control and CRC samples. The Shannon diversity was normalized to mean = 0 and standard deviation = 1. (PDF 606 kb) [file 40168_2018_451_MOESM1_ESM.pdf]

Streptococcus sp I-G2

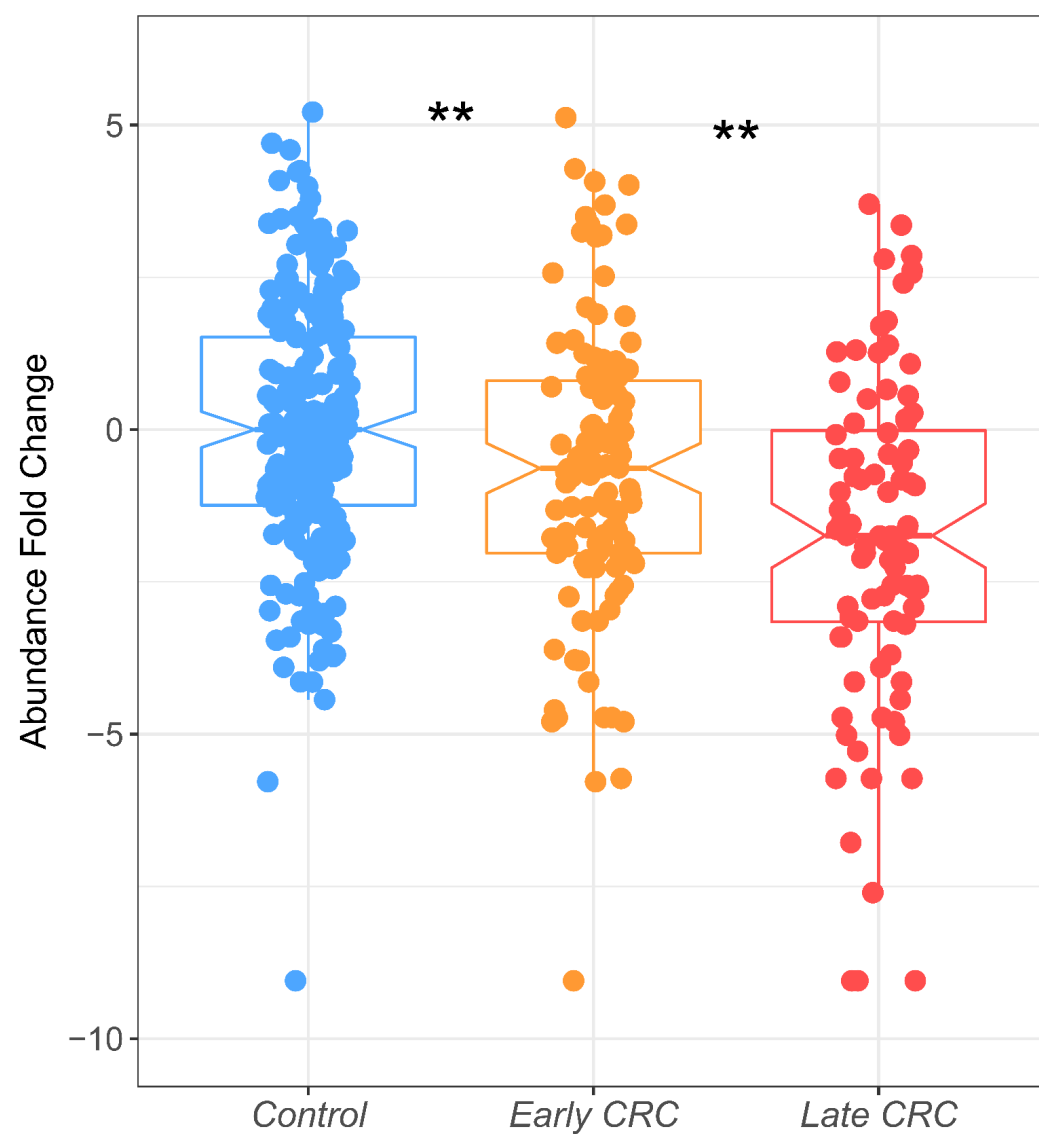

Shewanella woodyi

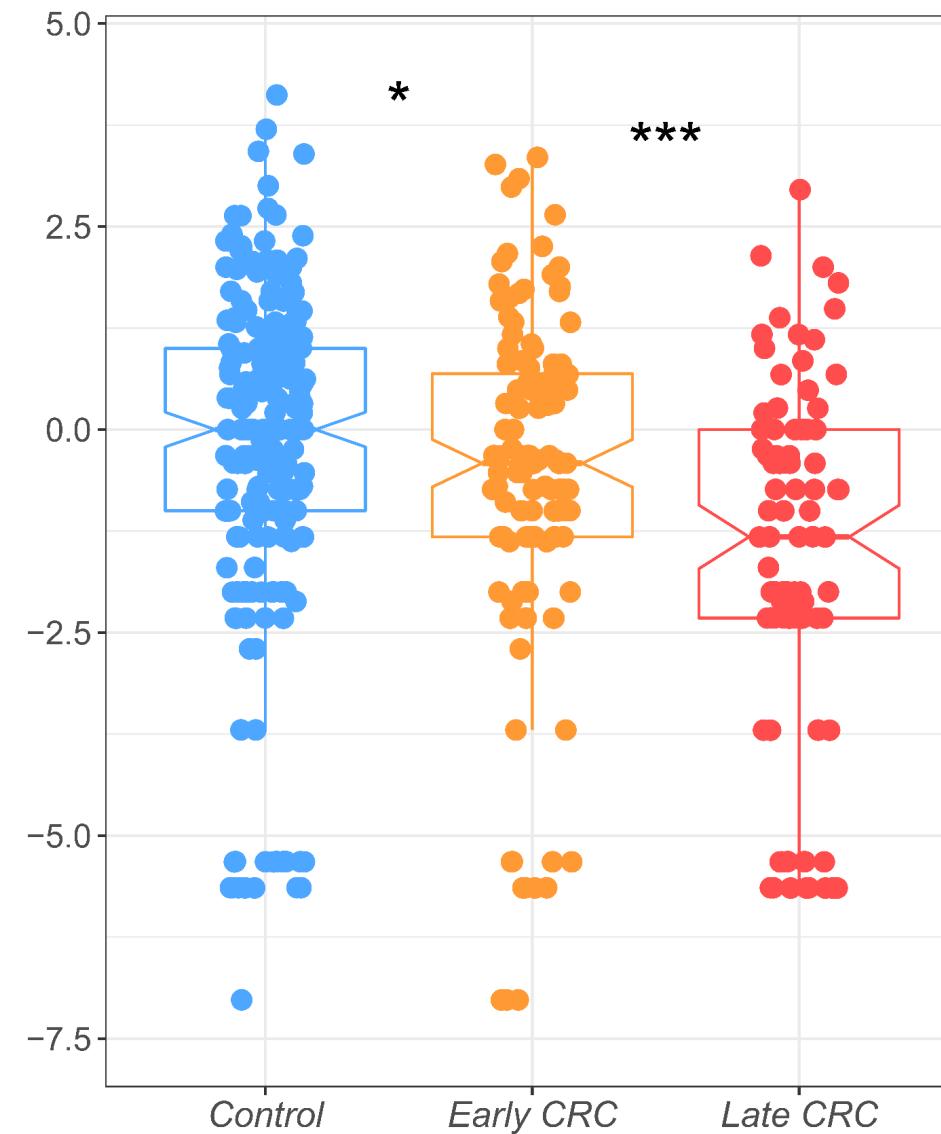

Mycoplasma penetrans

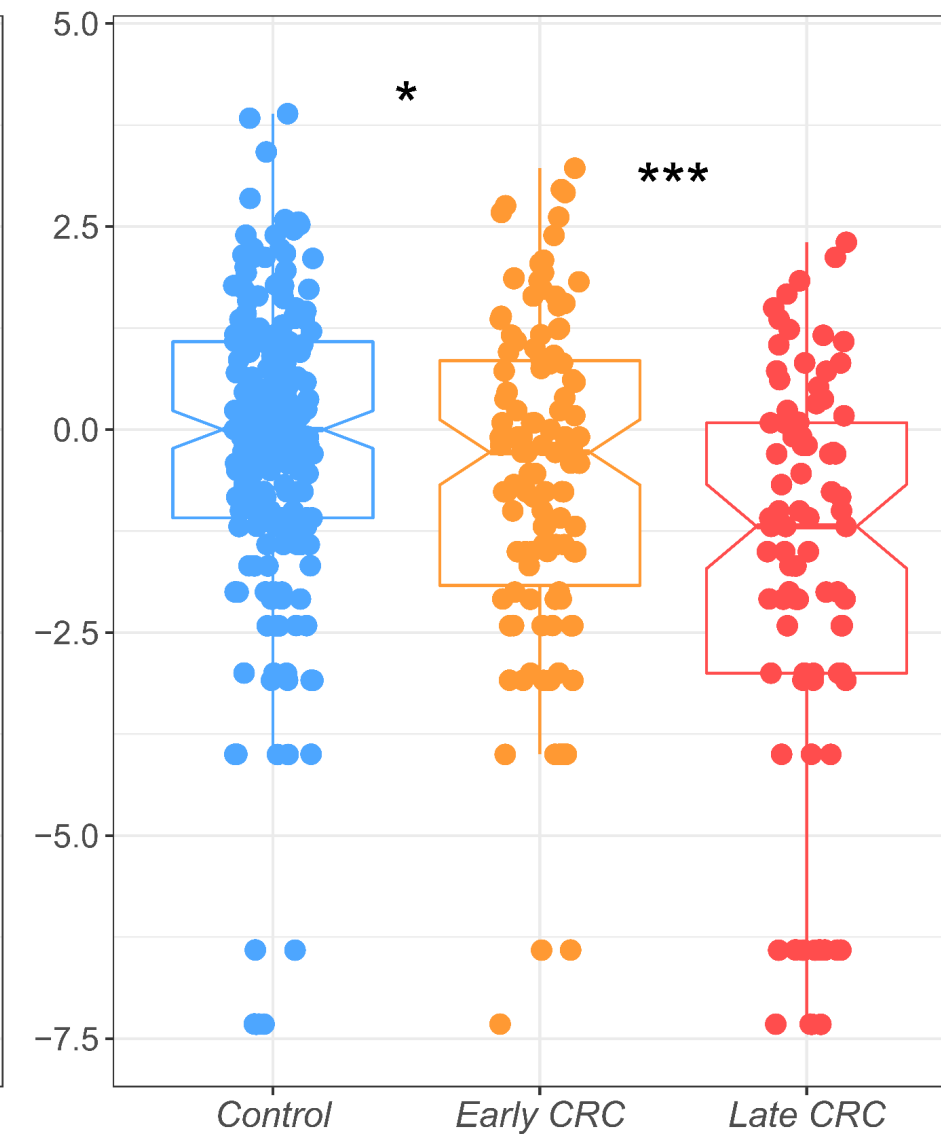

Control  
Early CRC  
Late CRC

Supplement: Supplementary file 6 — Figure S5. Boxplot of the three species whose abundance significantly decreased between control, early-, and late-stage CRC (P < 0.05, *P < 0.01, **P < 0.001, ***). (PDF 342 kb) [file 40168_2018_451_MOESM6_ESM.pdf]

**A**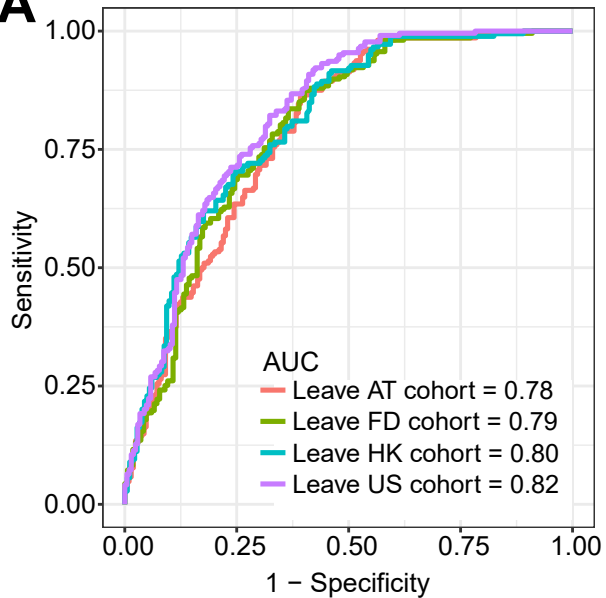**B**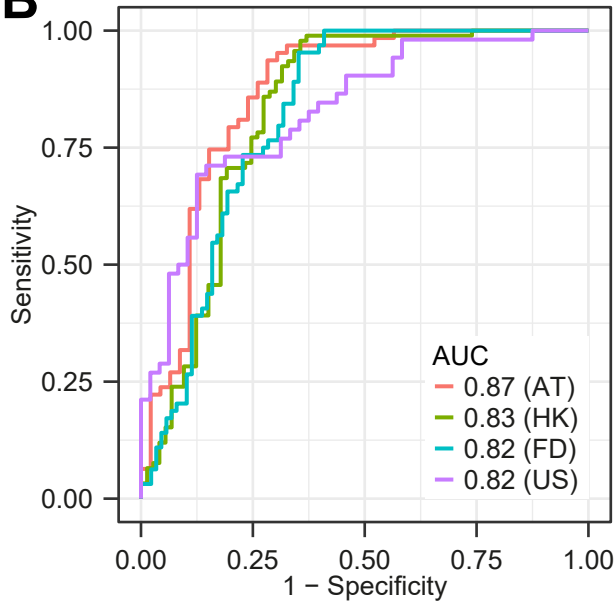

Supplement: Supplementary file 7 — Figure S3. (A) Prediction performance of seven CRC-enriched bacteria using a single model. (B) Prediction performance of the optimized the model of the individual cohort. (PDF 524 kb) [file 40168_2018_451_MOESM7_ESM.pdf]

**A**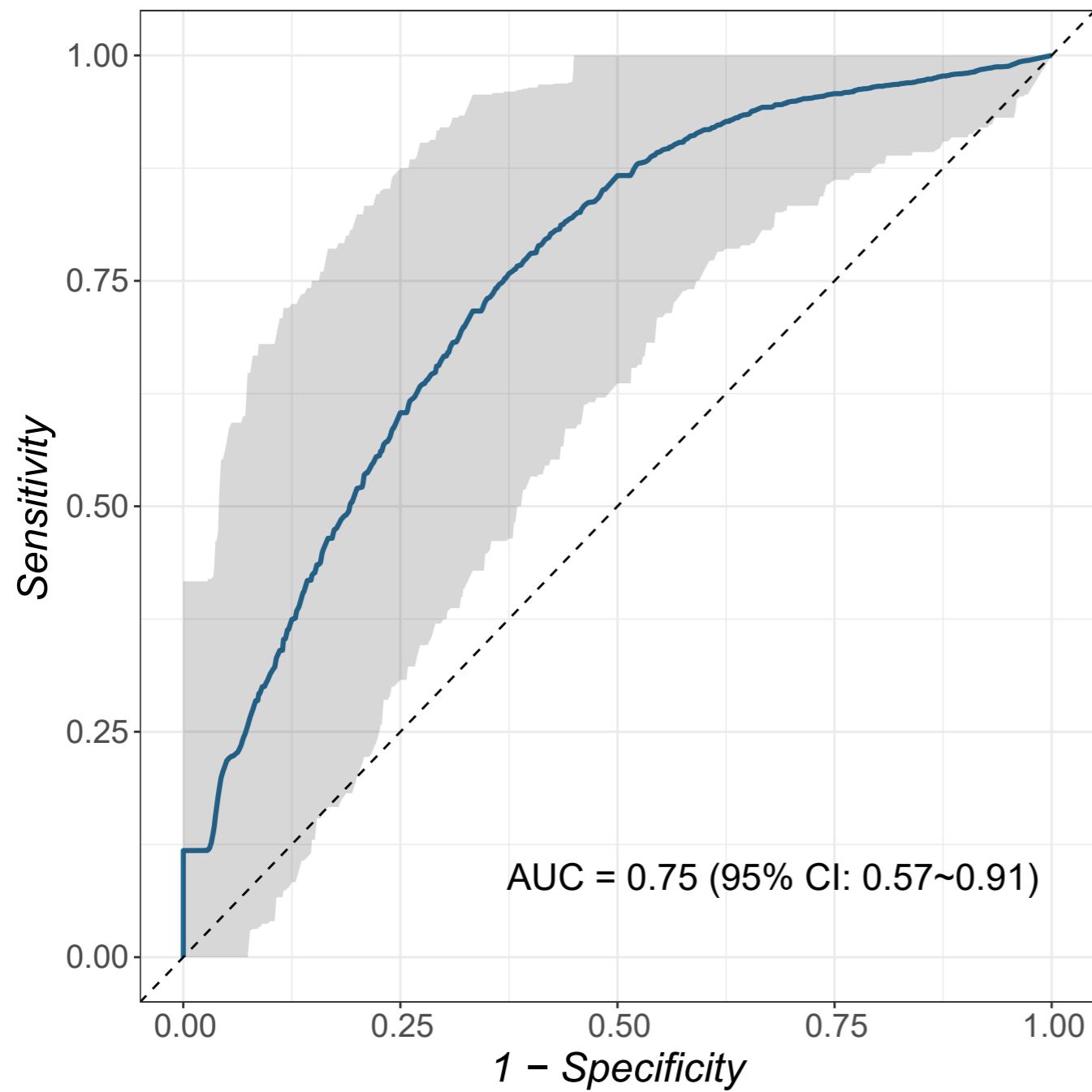**B**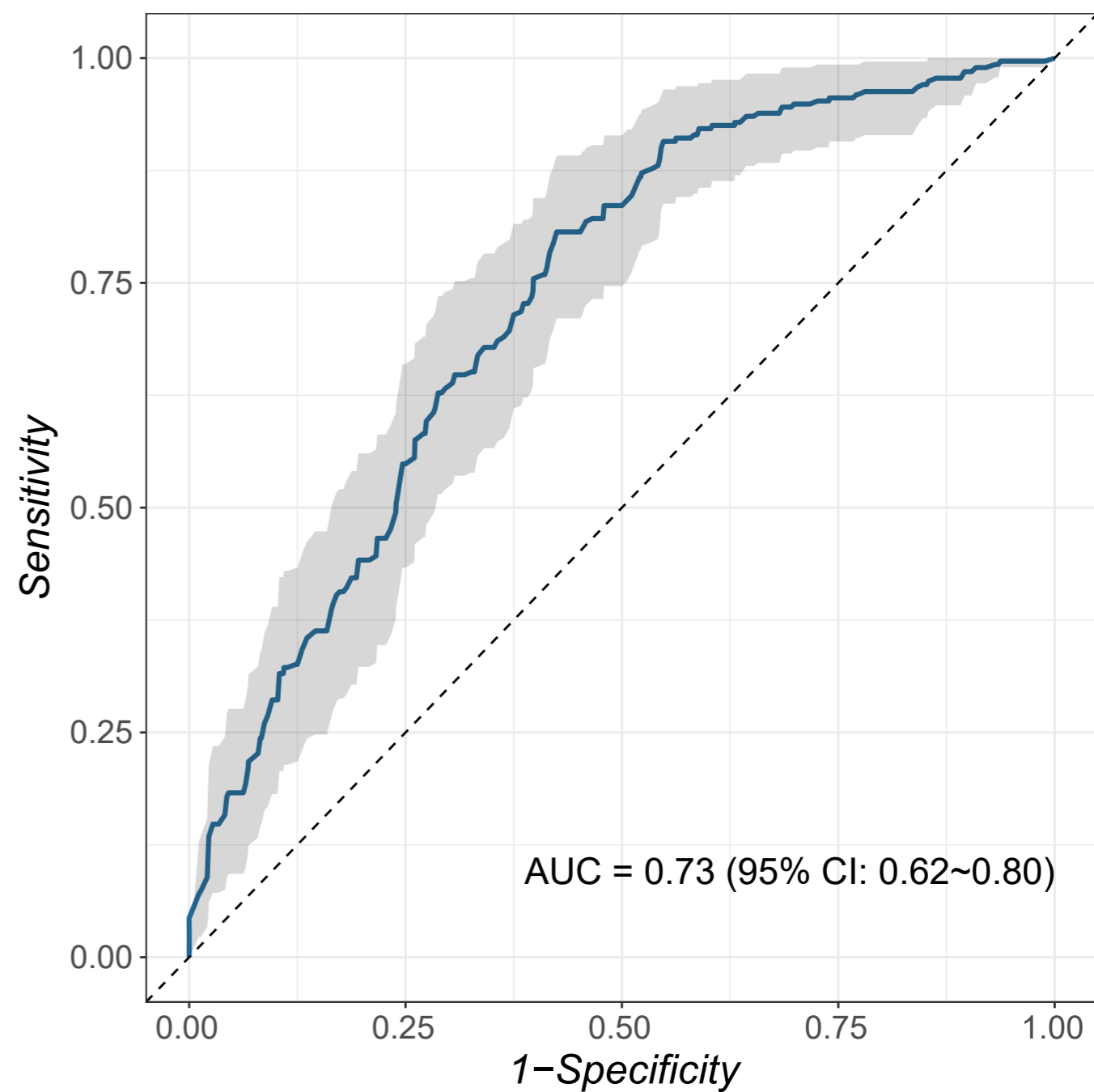

Supplement: Supplementary file 8 — Figure S12. (A) Prediction performance of the SVM model on the testing folds using 10-fold cross validation. (B) Prediction performance of the SVM model on the testing cohort using ‘leave-one-cohort-out’ approach. (PDF 125 kb) [file 40168_2018_451_MOESM8_ESM.pdf]

**A**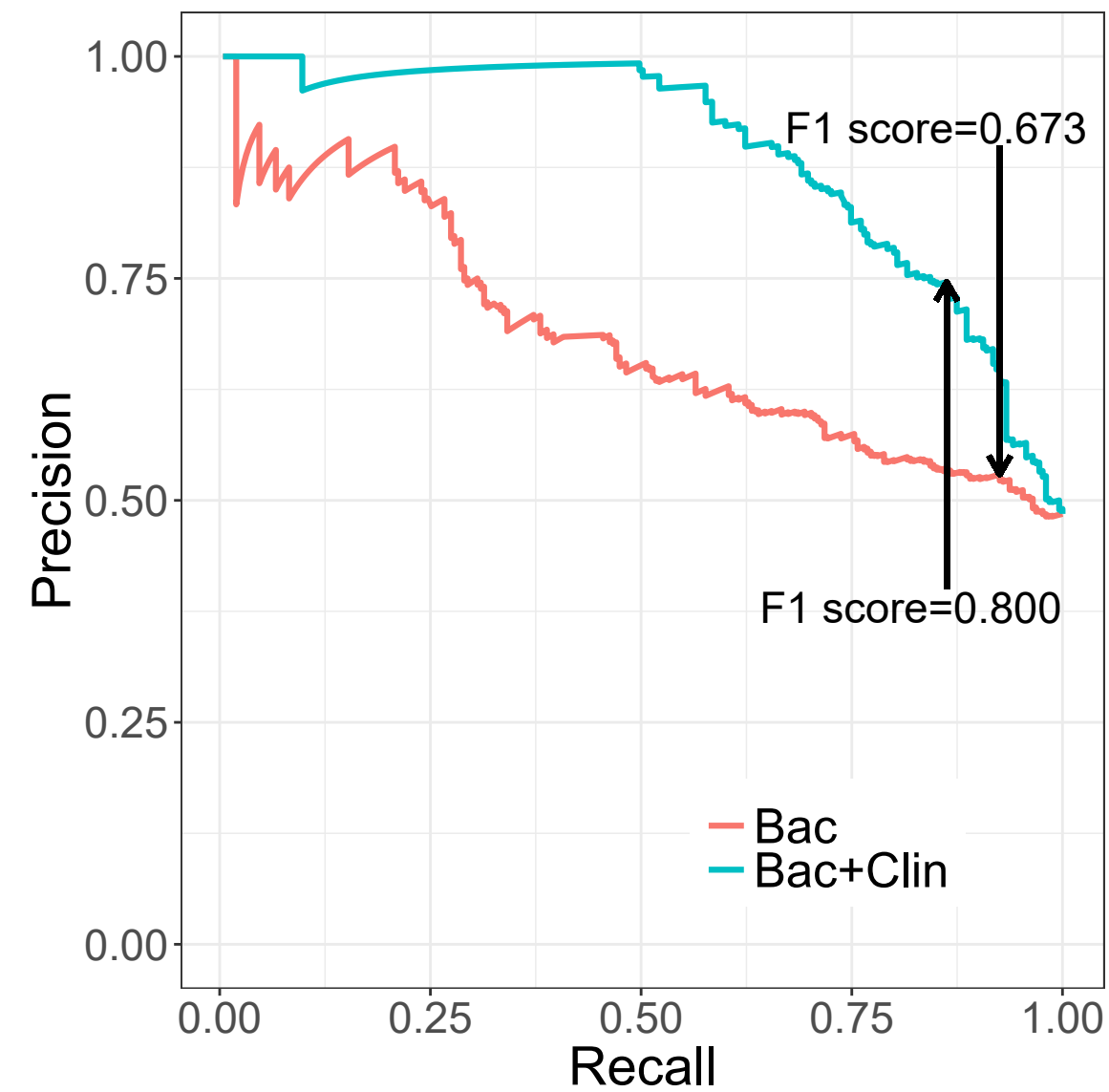**B**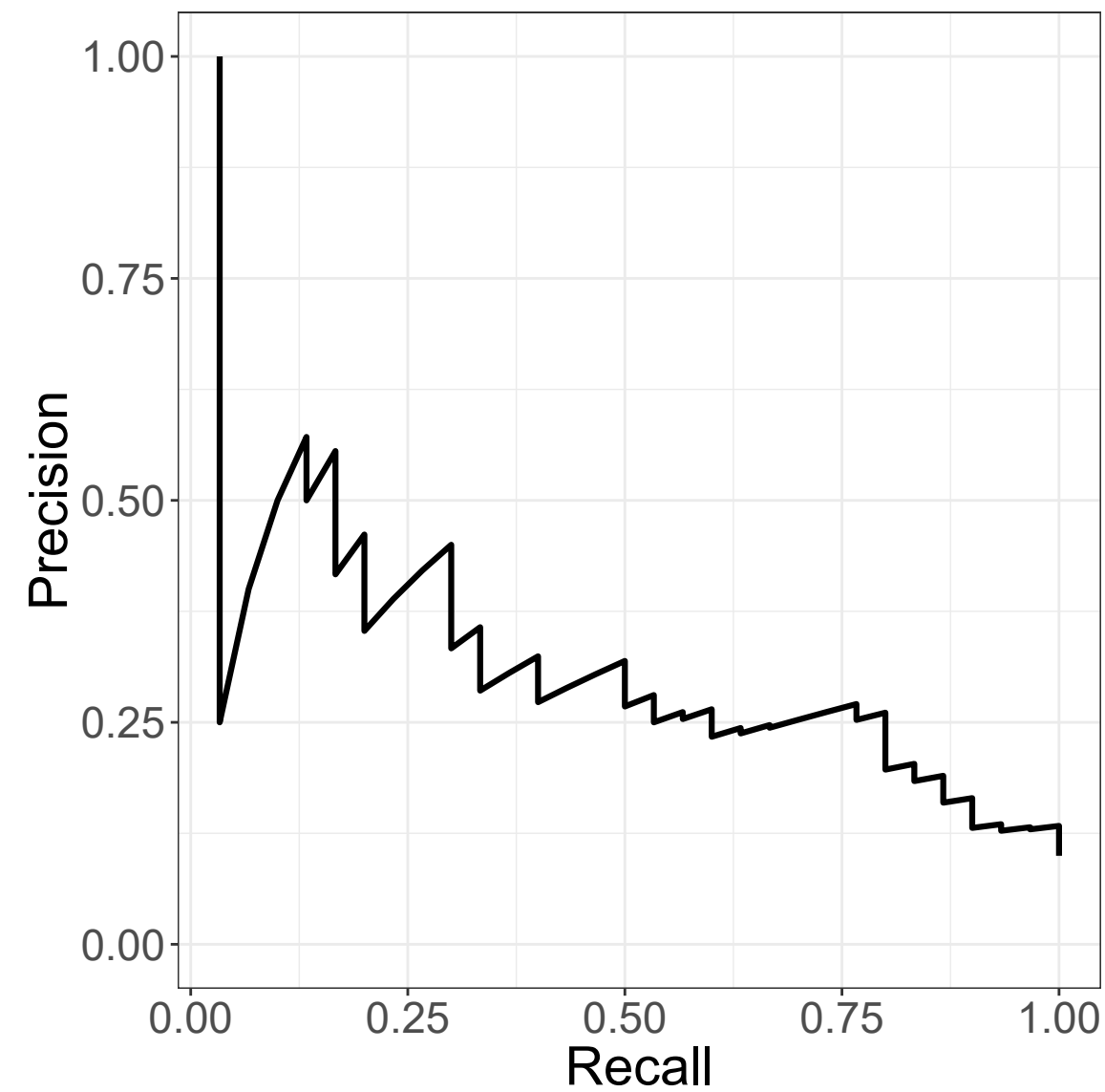**C**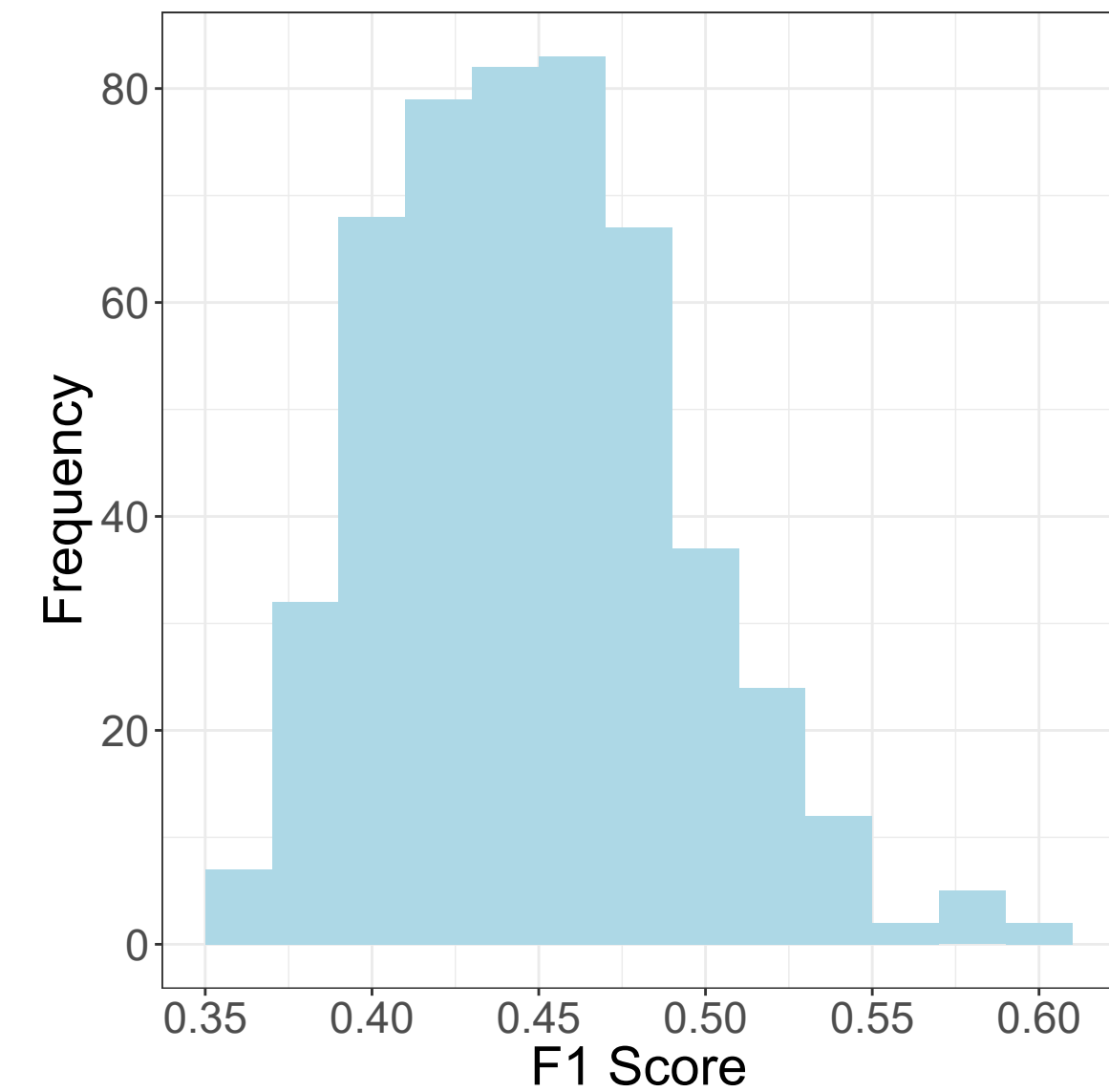

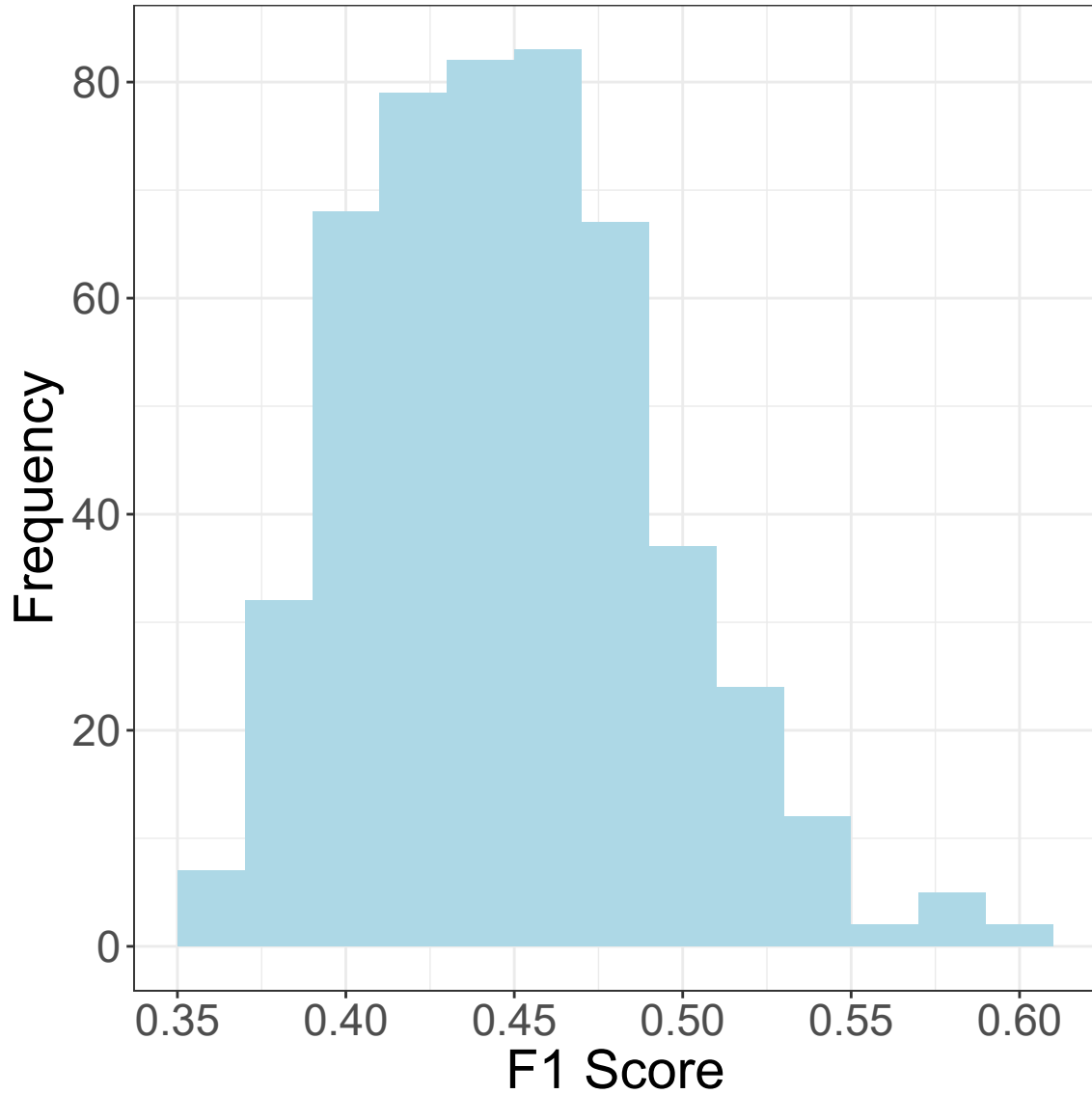

Supplement: Supplementary file 9 — Figure S4. (A) The precision and its corresponding recall index under different cut-off. The optimal F1 score was also provided. (B–C) With 271 controls and 30 cases, simulate probabilities with AUC≈0.80. Panel B shows a simulated precision and recall graph. Panel shows histogram of the optimal F1 scores with 500 simulations. (PDF 213 kb) [file 40168_2018_451_MOESM9_ESM.pdf]

**A**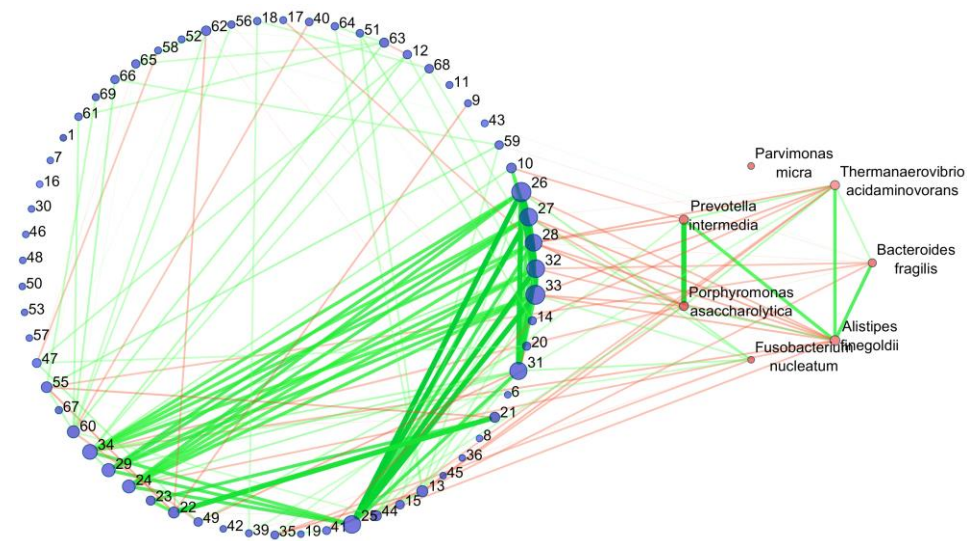**B**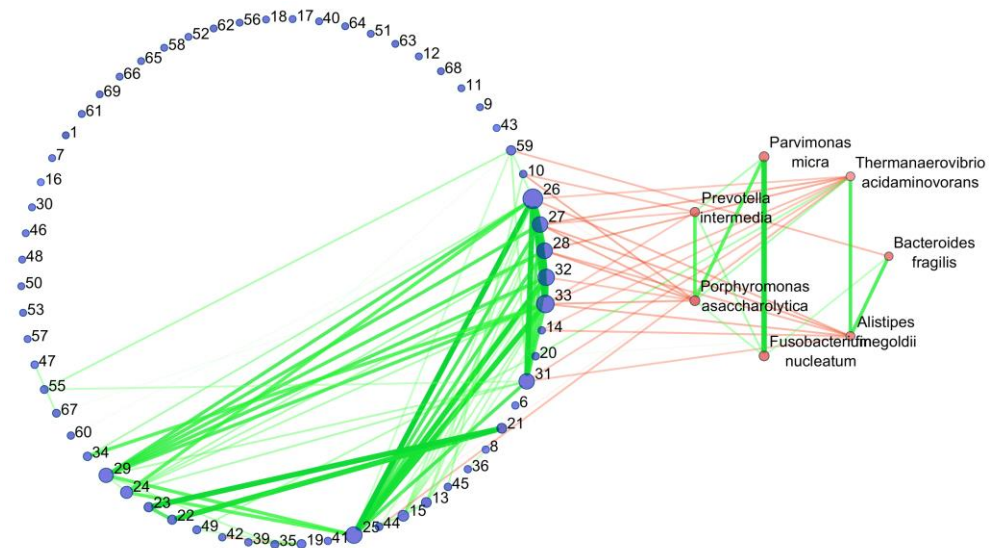

Supplement: Supplementary file 13 — Figure S7. (A) Correlation between the 69 CRC differentially abundant bacteria on CRC samples. The left circle includes the CRC-depleted bacteria and the right includes the CRC-enriched bacteria. (B) The correlation between the 69 CRC differentially abundant bacteria in control. Node attributes are attached in Additional file 15: Table S2 and Additional file 28: Table S3. (PDF 334 kb) [file 40168_2018_451_MOESM13_ESM.pdf]

**A**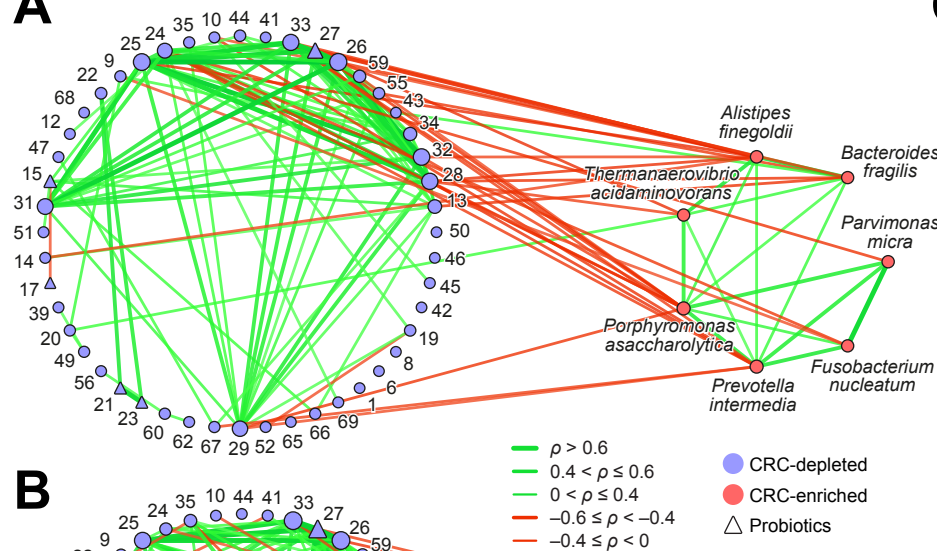**B**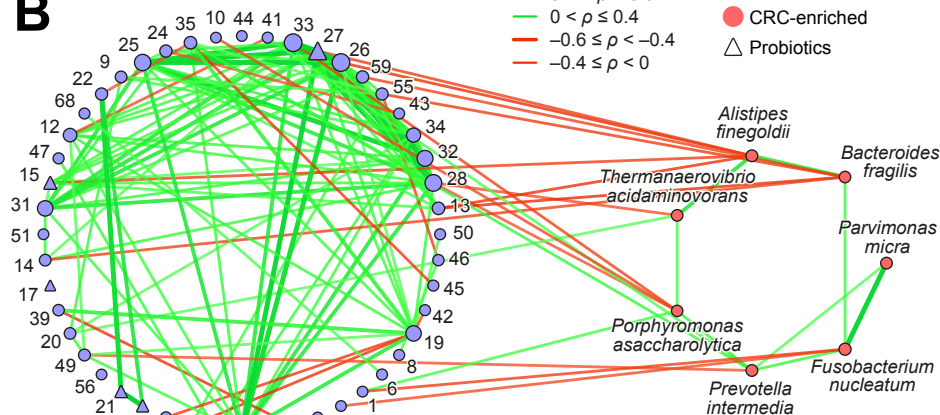**C**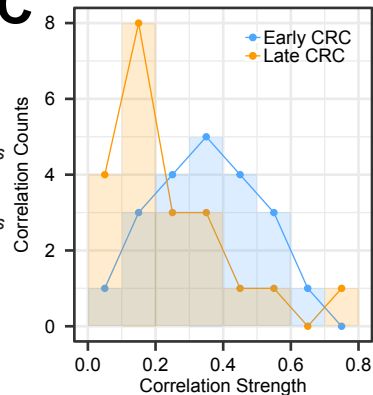

Supplement: Supplementary file 14 — Figure S9. (A)–(B) Correlations between the 69 differentially abundant bacteria in early- and late-stage CRC. Node attributes are attached in Additional file 4: Table S4 and Additional file 24: Table S5. (C) Comparison between the correlation networks among CRC-enriched bacteria in early- and late-stage CRC. (PDF 927 kb) [file 40168_2018_451_MOESM14_ESM.pdf]

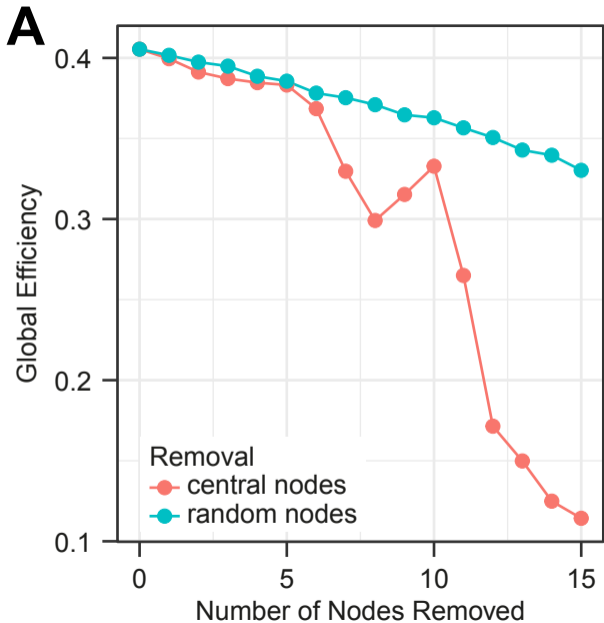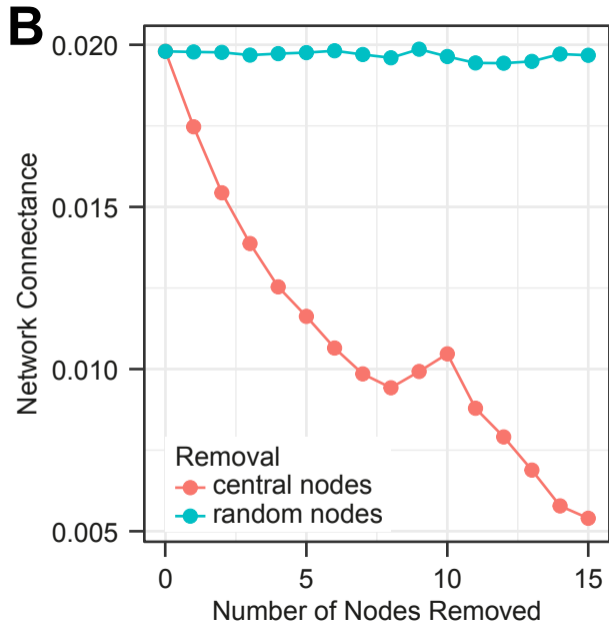

Supplement: Supplementary file 16 — Figure S8. Connectivity of the correlation network between the CRC-depleted bacteria in control samples. (PDF 367 kb) [file 40168_2018_451_MOESM16_ESM.pdf]

**A**

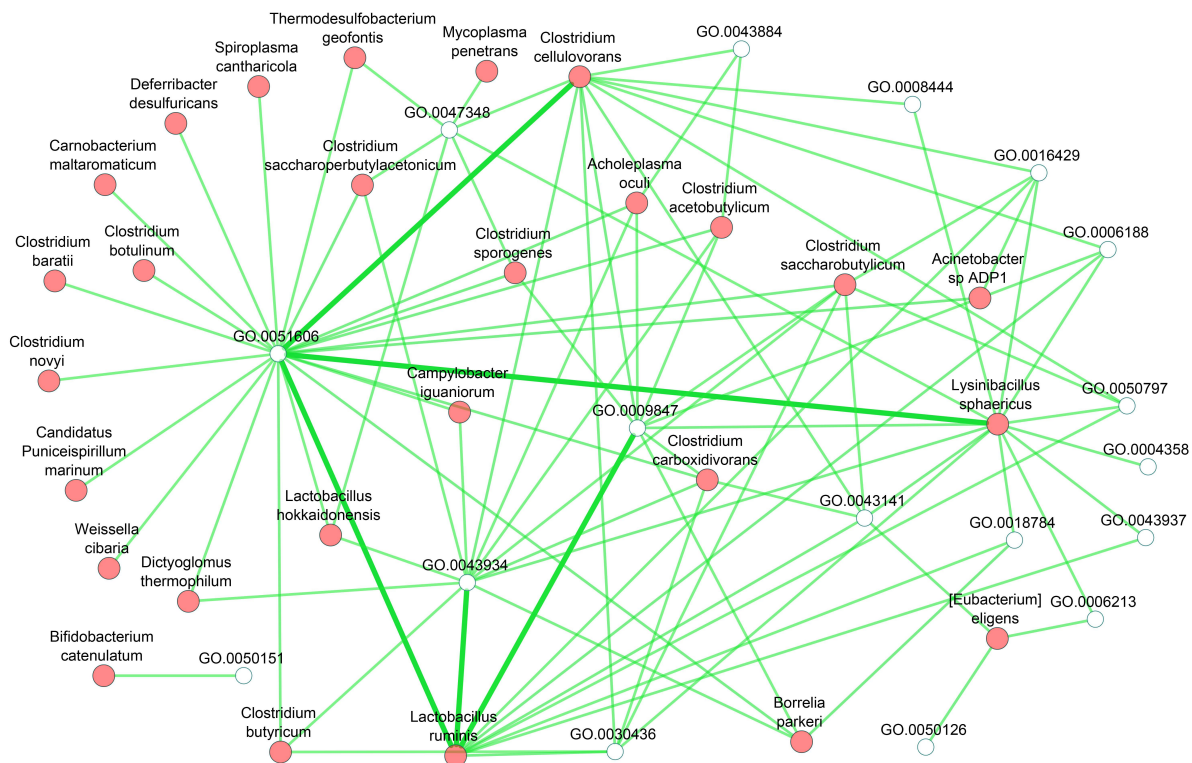

**B**

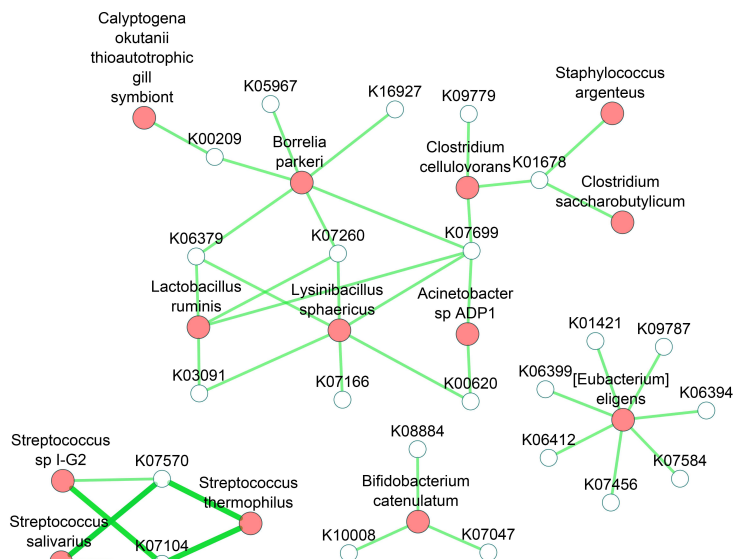

Supplement: Supplementary file 20 — Figure S11. Correlation network between CRC-depleted bacteria and CRC-depleted GO/KO categories (correlations with ρ > 0.5 were labeled in the figure). Node attributes are attached in Additional file 25: Table S11 and Additional file 26: Table S12. (PDF 3924 kb) [file 40168_2018_451_MOESM20_ESM.pdf]

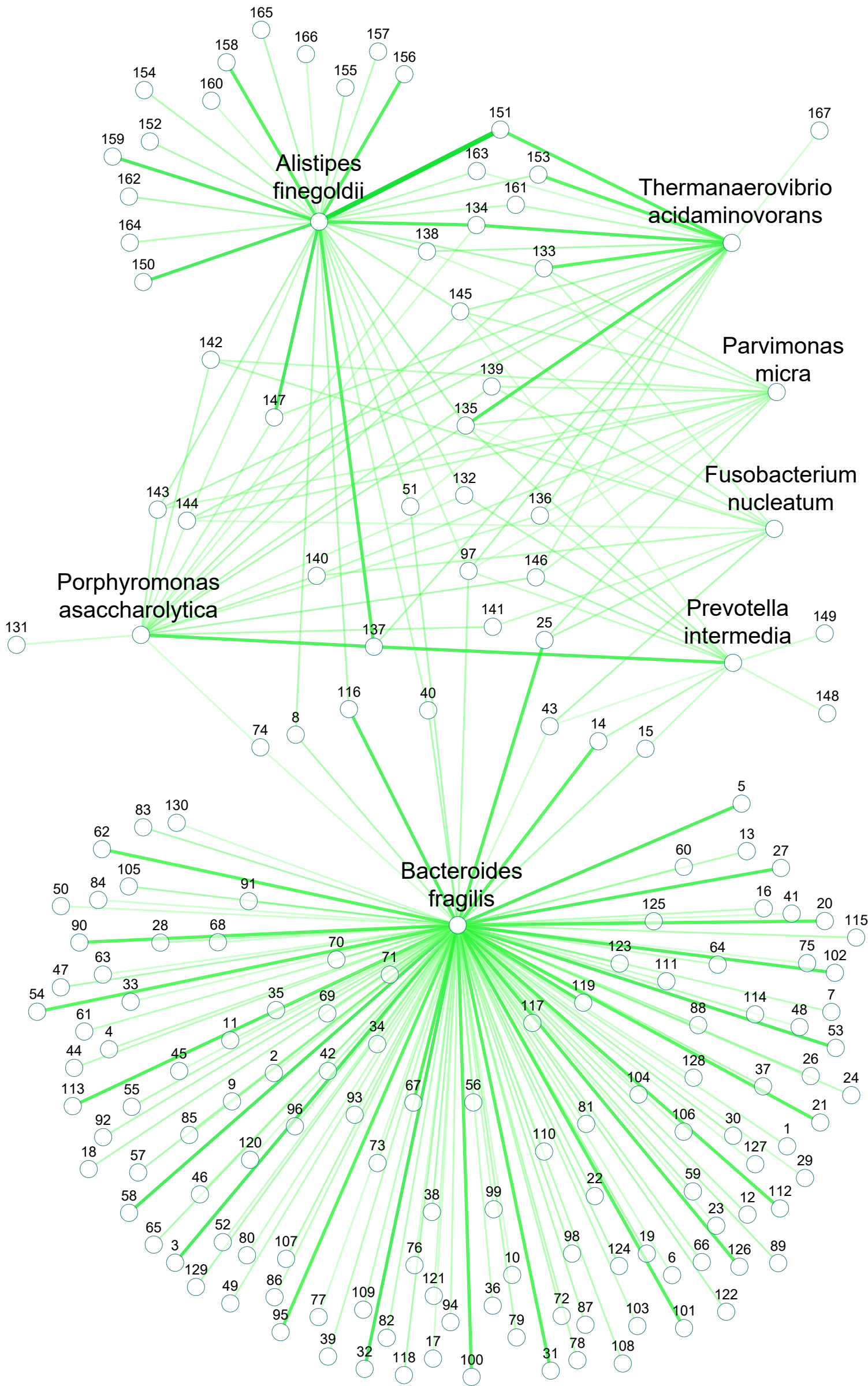

Supplement: Supplementary file 21 — Figure S10. Correlation network between CRC-enriched bacteria and CRC-enriched GO categories. Node attributes are attached in Additional file 27: Table S9. (PDF 376 kb) [file 40168_2018_451_MOESM21_ESM.pdf]

## Permutation Results

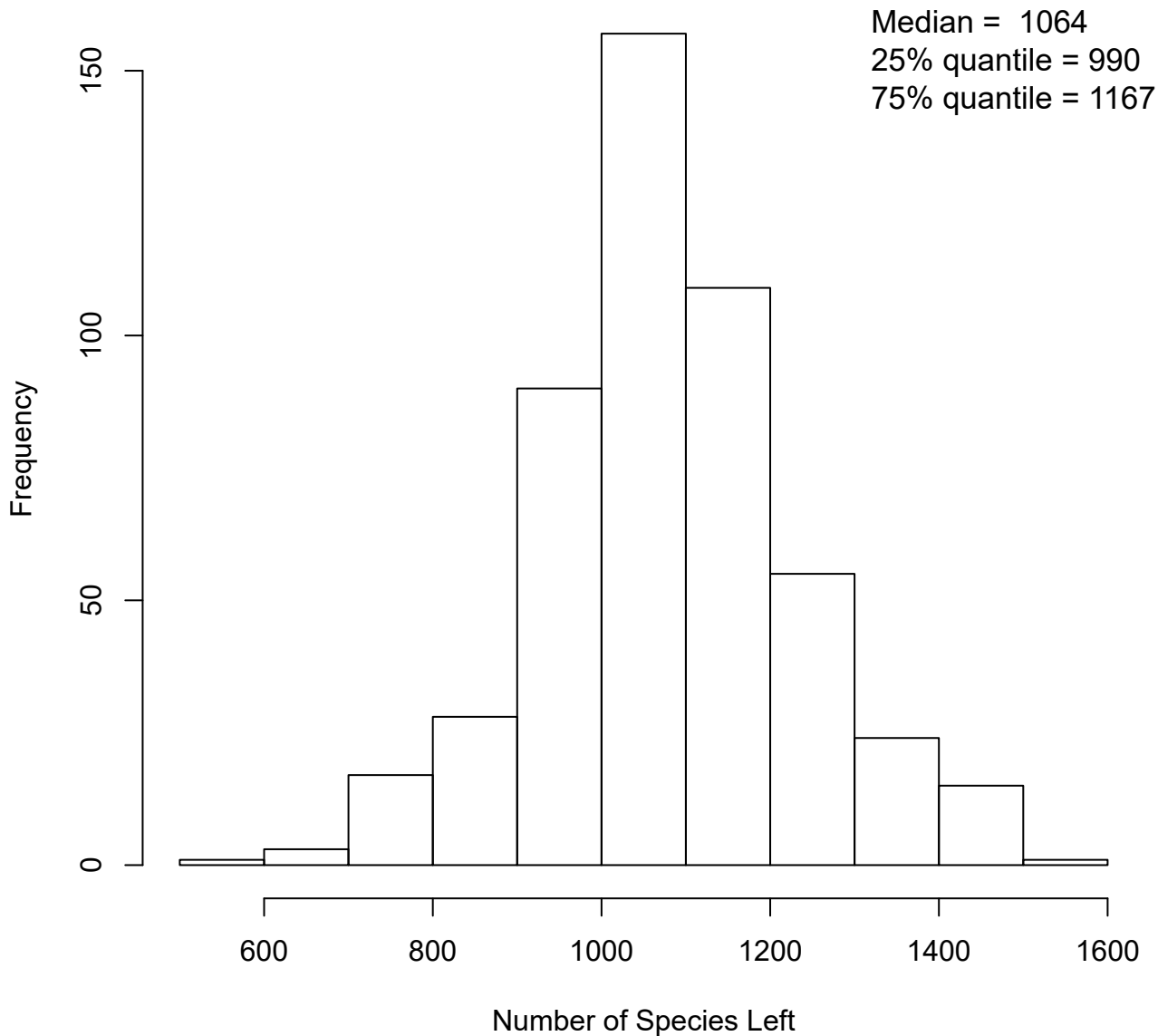

Supplement: Supplementary file 22 — Figure S13. Null distribution of the number of species left by removing the low abundant species, species missing in any cohort, and species with ‘divergent abundance change directions’. (PDF 117 kb) [file 40168_2018_451_MOESM22_ESM.pdf]

**A** Age distribution for Cohort C3

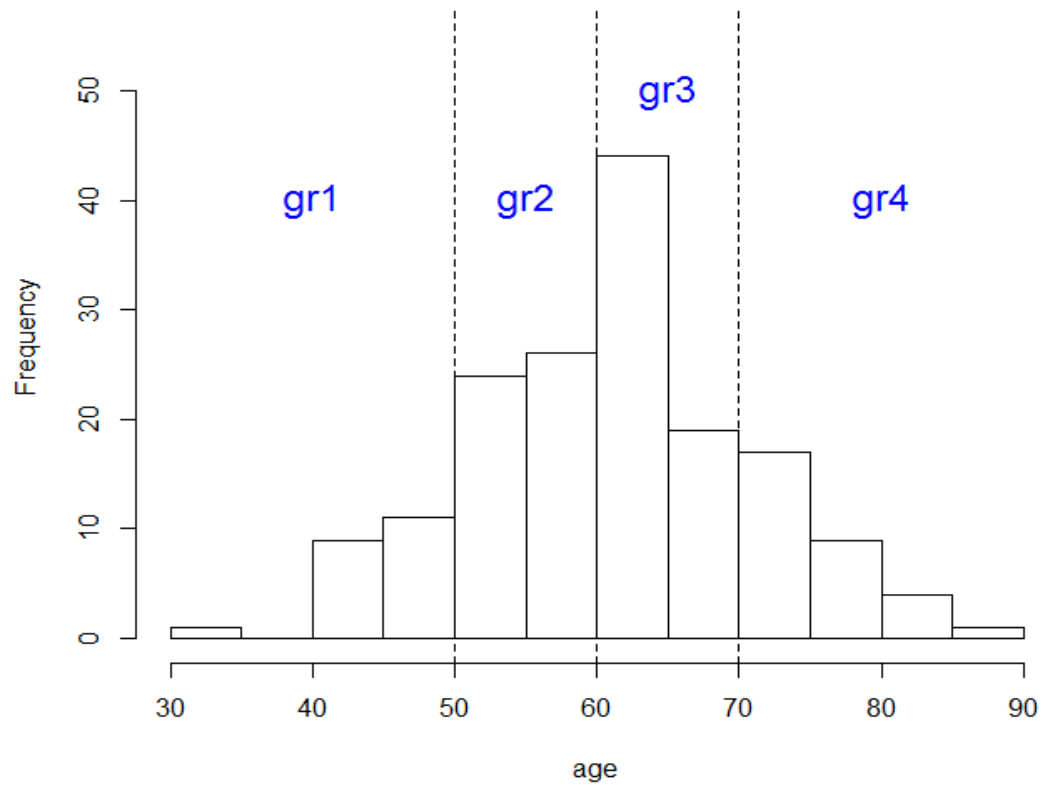

**B** Age distribution for Cohort C4

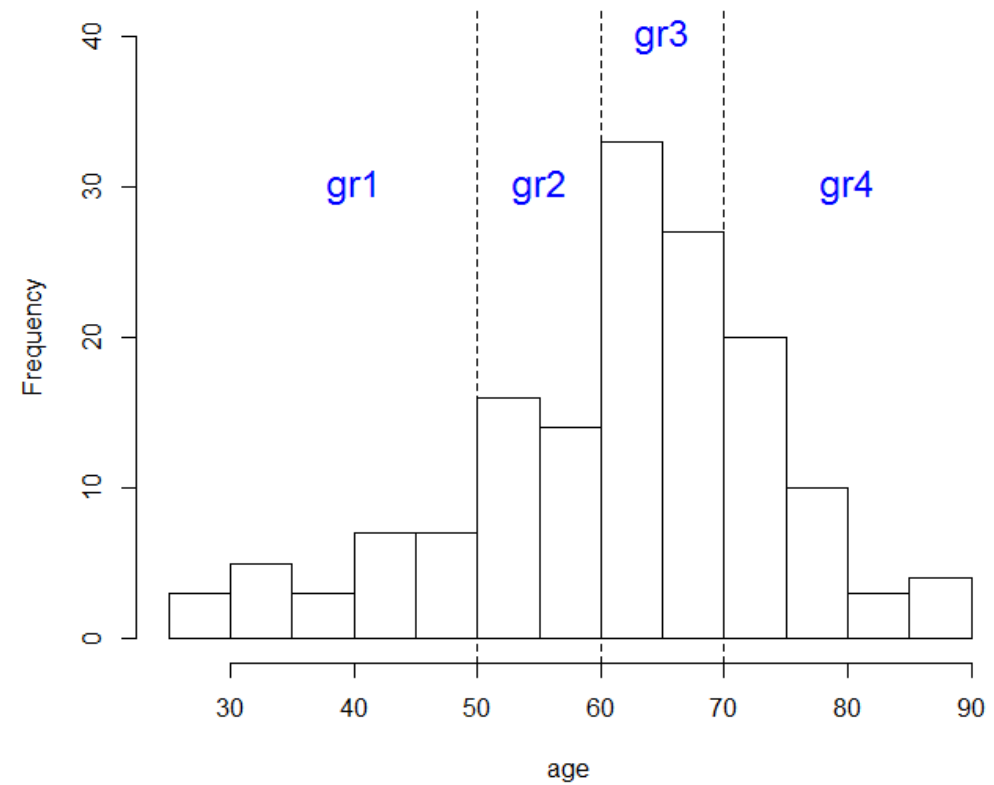

Supplement: Supplementary file 23 — Figure S2. Stratification of age in HK and FD cohorts. Conditioned Mann-Whitney U test was performed conditioned on the age strata. (PDF 214 kb) [file 40168_2018_451_MOESM23_ESM.pdf]
