# Supplementary material for: Multi-cohort analysis of colorectal cancer metagenome identified altered bacteria across populations and universal bacterial markers
Source: Microbiome. 2018 Apr 11;6:70. doi: 10.1186/s40168-018-0451-2 (PMC5896039; doi:10.1186/s40168-018-0451-2)
Supplement: Supplementary file 2 — Text Removing species with divergent abundance change directions. (DOCX 14 kb) [file 40168_2018_451_MOESM2_ESM.docx]

**Removing species with divergent abundance change directions**

We removed bacteria showing divergent abundance changes in our filtering pipeline. This is based our objective to identify species showing coherent changes in all populations, thereby maximizing the chance of them being genuinely related to CRC. In our project, we gave a point estimation for the bacteria abundance change direction using the median. We removed species showing a balanced discrepancy in abundance changes in the four cohorts (positive abundance changes in two cohorts and negative abundance changes in two other cohorts), or species showing significantly discrepant change in one or more cohorts. Statistical significance was defined by p<0.05 by the Mann-Whitney U test.

After removing the low abundant species (present in over 50% of the samples) and species that are missing in any cohort, we retained 1,732 species. Then, we filtered the species with divergent abundance change directions, and 994 species remained. We also explored the null distribution of the number of species left. We performed permutation to randomly assign the CRC/control labels in each cohort and removed species using the similar process. It turns out that around 1,000 species were left, close to our initial result where 994 species were left (Supplementary Figure 13). After filtering, we used the RankSum test to identify 7 CRC enriched and 62 CRC depleted bacterial species with pfp<0.01. We also demonstrated that, even without removing species that showed divergent abundance change directions, the identified species were still significantly differentially abundant in CRC. A total of 67 out of 69 differentially abundant species had pfp<0.01 whereas the two remaining species would have pfp<0.05 (Supplementary Table 14).
