# Supplementary material for: Multi-cohort analysis of colorectal cancer metagenome identified altered bacteria across populations and universal bacterial markers
Source: Microbiome. 2018 Apr 11;6:70. doi: 10.1186/s40168-018-0451-2 (PMC5896039; doi:10.1186/s40168-018-0451-2)
Supplement: Supplementary file 5 — Table S1. (DOCX 14 kb) [file 40168_2018_451_MOESM5_ESM.docx]

| **Cohort** | **AJCC Stage** | | | | | | **Lesion Location** | |
| --- | --- | --- | --- | --- | --- | --- | --- | --- |
|  | **Early Stage CRC** | | | **Late Stage CRC** | | **N/A** |  |  |
|  | **Stage 0** | **Stage I** | **Stage II** | **Stage III** | **Stage IV** |  | **Distal** | **Proximal** |
| **Cohort C1** | N/A | N/A | N/A | N/A | N/A | 48 | N/A | N/A |
| **Cohort C2** | 7 | 17 | 9 | 11 | 1 | 1 | 38 | 8 |
| **Cohort C3** | 0 | 18 | 22 | 25 | 8 | 0 | 60 | 13 |
| **Cohort C4** | 2 | 25 | 18 | 14 | 29 | 0 | 60 | 28 |
