# Supplementary material for: Multi-cohort analysis of colorectal cancer metagenome identified altered bacteria across populations and universal bacterial markers
Source: Microbiome. 2018 Apr 11;6:70. doi: 10.1186/s40168-018-0451-2 (PMC5896039; doi:10.1186/s40168-018-0451-2)

A

Species	pfp (4 cohorts)	pfp (3 cohorts)
Porphyromonas asaccharolytica	0	0
Fusobacterium nucleatum	0	0
Prevotella intermedia	0	0
Bacteroides fragilis	0	0.0025
Parvimonas micra	0	0.002
Thermanaerovibrio acidaminovorans	0.0029	0.0017
Alistipes finegoldii	0.0038	0.0233

B

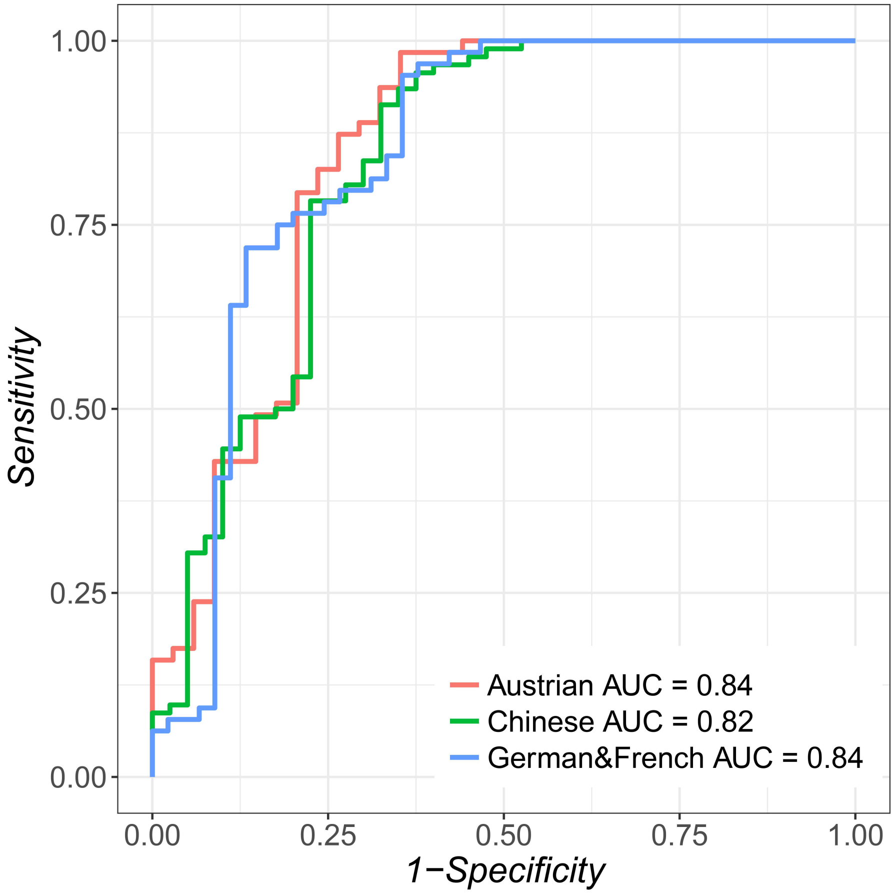

Supplement: Supplementary file 12 — Figure S6. (A) The table includes pfp of the seven CRC-enriched bacteria using all the four cohorts and three cohorts besides USA cohort. (B) Prediction performance of using seven CRC-enriched bacteria to classify early-stage CRC from control. (PDF 900 kb) [file 40168_2018_451_MOESM12_ESM.pdf]
